# Supplementary material for: The Developmental Shift of NMDA Receptor Composition Proceeds Independently of GluN2 Subunit-Specific GluN2 C-Terminal Sequences
Source: Cell Rep. 2018 Oct 23;25(4):841–851.e4. doi: 10.1016/j.celrep.2018.09.089 (PMC6218242; doi:10.1016/j.celrep.2018.09.089)
Supplement: Document S1. Figures S1–S3 [file mmc1.pdf]

Cell Reports, Volume *25*

Supplemental Information

The Developmental Shift of NMDA Receptor Composition Proceeds Independently of GluN2 Subunit-Specific GluN2 C-Terminal Sequences

Sean McKay, Tomás J. Ryan, Jamie McQueen, Tim Indersmitten, Katie F.M. Marwick, Philip Hasel, Maksym V. Kopanitsa, Paul S. Baxter, Marc-André Martel, Peter C. Kind, David J.A. Wyllie, Thomas J. O'Dell, Seth G.N. Grant, Giles E. Hardingham, and Noboru H. Komiyama

Supplemental Figures

Fig. S1

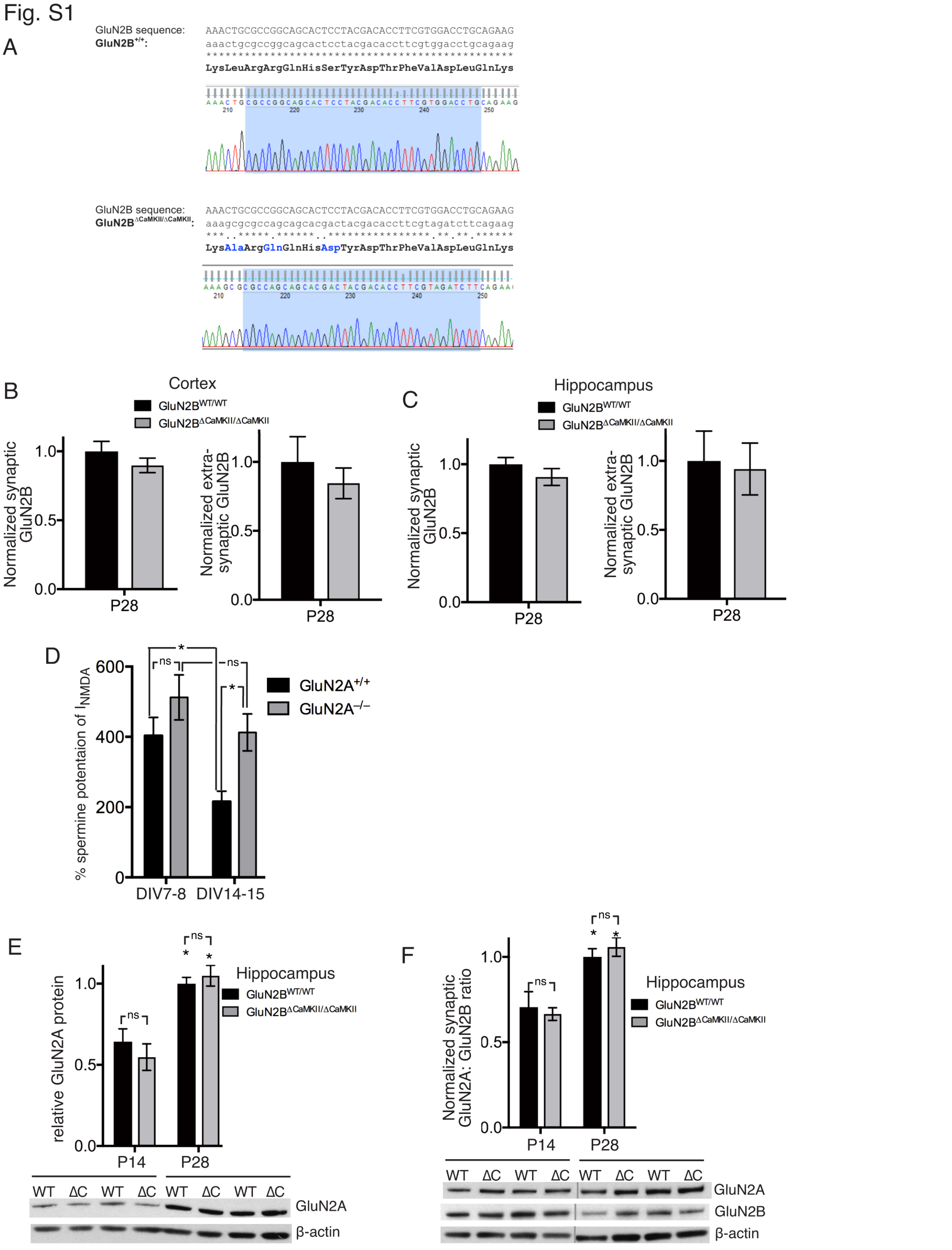

Fig. S1, relating to Figure 1. A) Sequencing data showing the mutations knocked into the *Grin2b* gene in the GluN2B$^{\Delta CaMKII/\Delta CaMKII}$ mouse line. **B)** Synaptic (left) and extrasynaptic (right) GluN2B protein expression was measured in P28 neocortices of GluN2B$^{\Delta CaMKII/\Delta CaMKII}$ (n=8) and GluN2B$^{+/+}$ mice (n=8). **C)** Synaptic (left) and extrasynaptic (right) GluN2B expression was measured in P28

hippocampi of GluN2B$^{\Delta CaMKII/\Delta CaMKII}$ (n=8) and GluN2B$^{+/+}$ mice (n=8). **D)** Developmental shift in spermine potentiation of NMDAR currents requires GluN2A. Percentage potentiation of NMDAR currents by spermine (200 µM) was measured at the indicated stages for GluN2A$^{+/+}$ and GluN2A$^{-/-}$ rat neurons. **P*<0.05 compared to DIV7-8 of the same genotype, 2-way ANOVA plus Sidak's post-hoc test (n=12 of all ages/genotypes). **E)** Whole neocortical extracts from P14 and P28 mice of the indicated genotype were analysed for GluN2A expression, normalized to ß-actin. **P*<0.05 compared to P14 of the same genotype, 2-way ANOVA plus Sidak's post-hoc test (n=8). Lower panel shows example blot. **F)** Post-synaptic density (PSD) extracts from P14 and P28 mice of the indicated genotype were analysed for GluN2A and GluN2B expression, normalized to ß-actin, and the ratio calculated. **P*<0.05 compared to P14 of the same genotype, 2-way ANOVA plus Sidak's post-hoc test (n=8). Lower panel shows example blot.

Fig. S2

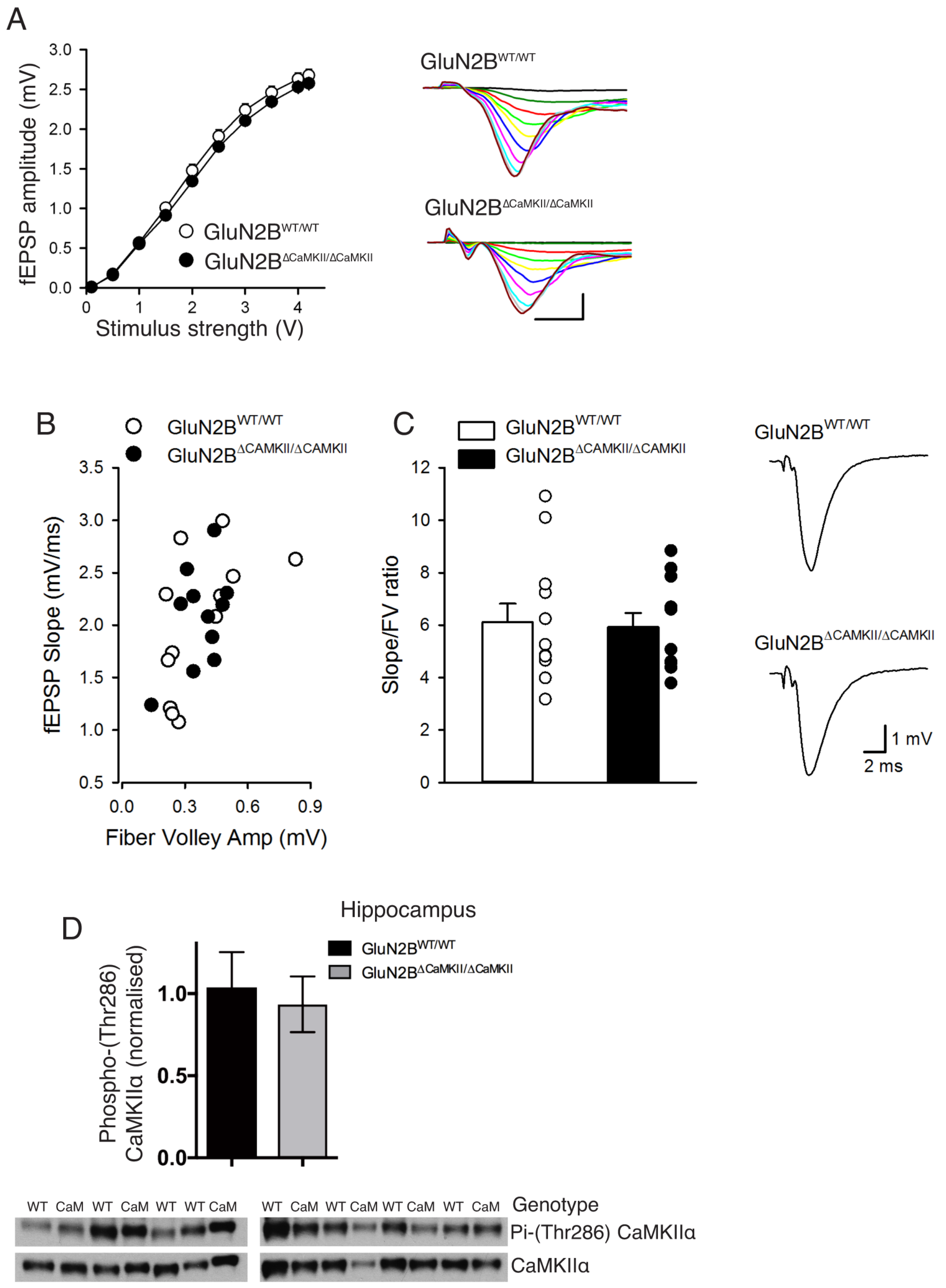

Fig. S2, relating to Figure 2. A) Input-output relationships illustrate averaged fEPSP amplitudes in slices from GluN2B$^{\Delta CaMKII/\Delta CaMKII}$ (35 slices from n=11 mice) and GluN2B$^{WT/WT}$ mice (29 slices from n=9 mice) in response to stimulation of Schaffer collaterals by biphasic voltage pulses of 0.1 – 4.2 V. Right panel shows representative families of fEPSP traces. Scale bar: 1 mV/2 ms. **B-C)** A comparison of fEPSP slopes and presynaptic fiber volley amplitudes for 12 slices from 5 GluN2B$^{WT/WT}$ mice and 11 slices from 6 GluN2B GluN2B$^{\Delta CaMKII/\Delta CaMKII}$ mice. **D)** Phospho-T286 CaMKIIα levels in PSD fractions of P28 GluN2B$^{\Delta CaMKII/\Delta CaMKII}$ vs GluN2B$^{WT/WT}$ hippocampi were analysed by western blot, normalising to total CaMKIIα.

Fig. S3

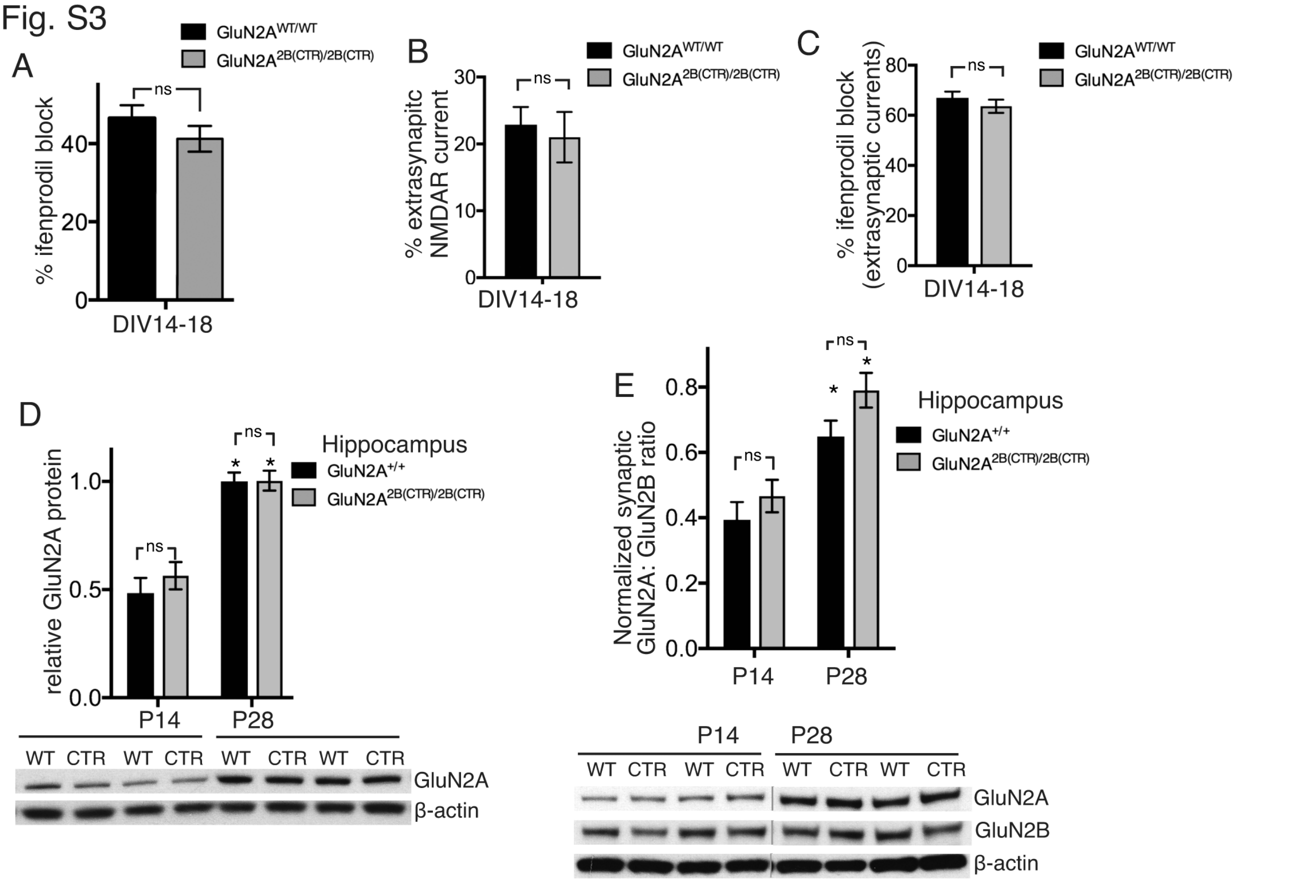

Fig. S3, relating to Figure 3. A) Ifenprodil sensitivity of NMDAR currents was measured at DIV14-18 in neurons of the indicated genotypes. GluN2A$^{WT/WT}$: n=24 cells; GluN2A$^{2B(CTR)/2B(CTR)}$: n=30 cells. **B,C)** Extrasynaptic NMDAR currents were calculated and expressed as a percentage of whole-cell currents (B) and their sensitivity to ifenprodil (3 μM) was measured in DIV14-18 neurons, an age where significant GluN2A is expressed. N=12 of both genotypes. **D)** Whole hippocampal extracts from P14 and P28 mice of the indicated genotype were analysed for GluN2A expression, normalized to ß-actin. **P*<0.05 compared to P14 of the same genotype, 2-way ANOVA plus Sidak's post-hoc test (n=8). **E)** Post-synaptic density (PSD) extracts from P14 and P28 hippocampi of the indicated genotype were analysed for GluN2A and GluN2B expression, normalized to ß-actin, and the ratio calculated. **P*<0.05 compared to P14 of the same genotype, 2-way ANOVA plus Sidak's post-hoc test (n=8).
