# Supplementary material for: The Developmental Shift of NMDA Receptor Composition Proceeds Independently of GluN2 Subunit-Specific GluN2 C-Terminal Sequences
Source: Cell Rep. 2018 Oct 23;25(4):841–851.e4. doi: 10.1016/j.celrep.2018.09.089 (PMC6218242; doi:10.1016/j.celrep.2018.09.089)
Supplement: Document S2. Article plus Supplemental Information [file mmc2.pdf]

Cell Reports

Report

The Developmental Shift of NMDA Receptor Composition Proceeds Independently of GluN2 Subunit-Specific GluN2 C-Terminal Sequences

Graphical Abstract

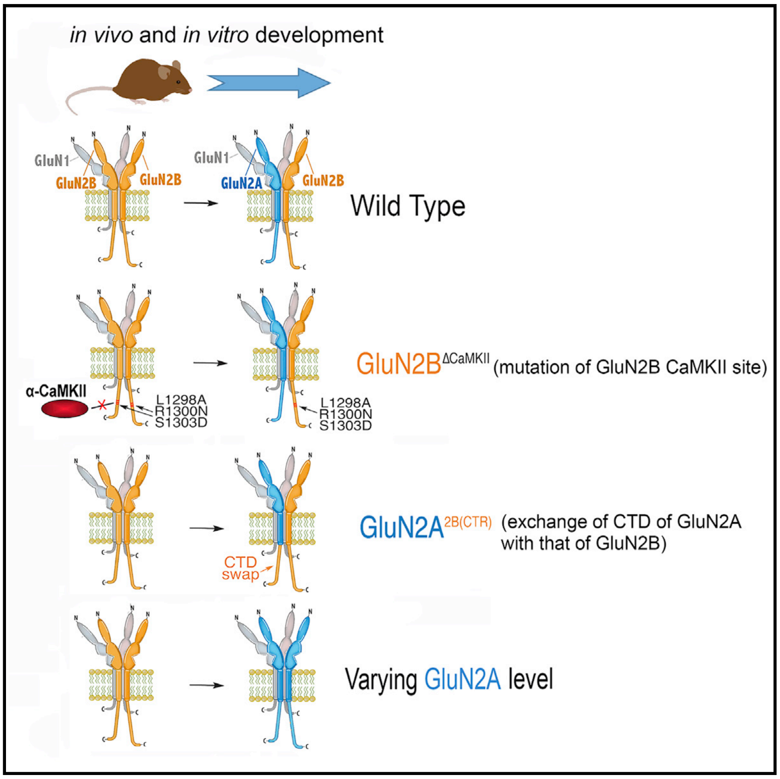

Highlights

- Mutating the GluN2B CaMKII site affects phosphorylation of its C-terminal domain
- The developmental changes in NMDAR composition and synaptogenesis occur normally
- Changes in NMDAR composition do not require distinct GluN2 C-terminal domains
- Developmental changes in NMDAR composition are primarily sensitive to GluN2A levels

Authors

Sean McKay, Tomás J. Ryan, Jamie McQueen, ..., Seth G.N. Grant, Giles E. Hardingham, Noboru H. Komiyama

Correspondence

giles.hardingham@ed.ac.uk (G.E.H.), n.komiyama@ed.ac.uk (N.H.K.)

In Brief

An important milestone in forebrain neuronal development is the switch in composition of the NMDA receptor: GluN2A becomes incorporated into previously GluN2B-dominated NMDARs. Using knockin mice, McKay et al. find that, contrary to earlier proposed models, the switch does not require GluN2A and GluN2B to possess distinct C-terminal domain sequences.

McKay et al., 2018, Cell Reports *25*, 841–851
October 23, 2018 © 2018 The Author(s).
https://doi.org/10.1016/j.celrep.2018.09.089

CellPress

OPEN ACCESS
CellPress

The Developmental Shift of NMDA Receptor Composition Proceeds Independently of GluN2 Subunit-Specific GluN2 C-Terminal Sequences

Sean McKay,[1,2,3,10] Tomás J. Ryan,[4,5,10] Jamie McQueen,[1,2,3,10] Tim Indersmitten,[6] Katie F.M. Marwick,[1,2] Philip Hasel,[1,2,3] Maksym V. Kopanitsa,[7,8] Paul S. Baxter,[1,2,3] Marc-André Martel,[1] Peter C. Kind,[1,2] David J.A. Wyllie,[1,2] Thomas J. O'Dell,[6] Seth G.N. Grant,[2,7,9] Giles E. Hardingham,[1,2,3,11,*] and Noboru H. Komiyama[2,7,9,*]

[1]Centre for Discovery Brain Sciences, University of Edinburgh, Hugh Robson Building, George Square, Edinburgh EH8 9XD, UK
[2]Simons Initiative for the Developing Brain, University of Edinburgh, Hugh Robson Building, George Square, Edinburgh EH8 9XD, UK
[3]UK Dementia Research Institute at the University of Edinburgh, Chancellor's Building, Edinburgh Medical School, Edinburgh EH16 4SB, UK
[4]School of Biochemistry and Immunology, Trinity Biomedical Sciences Institute and Trinity College Institute of Neuroscience, Trinity College Dublin, Dublin, Ireland
[5]Florey Institute of Neuroscience and Mental Health, Melbourne Brain Centre, University of Melbourne, Parkville, VIC, Australia
[6]Department of Physiology, David Geffen School of Medicine, University of California, Los Angeles, Los Angeles, CA 90095, USA
[7]The Wellcome Trust Sanger Institute, Hinxton CB10 1SA, UK
[8]UK Dementia Research Institute at Imperial College London, Hammersmith Hospital Campus, Imperial College, London W12 0NN, UK
[9]Centre for Clinical Brain Sciences, University of Edinburgh Chancellor's Building, Edinburgh, UK
[10]These authors contributed equally
[11]Lead Contact
*Correspondence: giles.hardingham@ed.ac.uk (G.E.H.), n.komiyama@ed.ac.uk (N.H.K.)
https://doi.org/10.1016/j.celrep.2018.09.089

SUMMARY

The GluN2 subtype (2A versus 2B) determines biophysical properties and signaling of forebrain NMDA receptors (NMDARs). During development, GluN2A becomes incorporated into previously GluN2B-dominated NMDARs. This "switch" is proposed to be driven by distinct features of GluN2 cytoplasmic C-terminal domains (CTDs), including a unique CaMKII interaction site in GluN2B that drives removal from the synapse. However, these models remain untested in the context of endogenous NMDARs. We show that, although mutating the endogenous GluN2B CaMKII site has secondary effects on GluN2B CTD phosphorylation, the developmental changes in NMDAR composition occur normally and measures of plasticity and synaptogenesis are unaffected. Moreover, the switch proceeds normally in mice that have the GluN2A CTD replaced by that of GluN2B and commences without an observable decline in GluN2B levels but is impaired by GluN2A haploinsufficiency. Thus, GluN2A expression levels, and not GluN2 subtype-specific CTD-driven events, are the overriding factor in the developmental switch in NMDAR composition.

INTRODUCTION

NMDARs (N-methyl-D-aspartate [NMDA] receptors) are glutamate-gated cation channels with high Ca^{2+} permeability that play key roles in CNS processes, such as synaptic transmission, learning and memory, development, neuroprotective signaling, and redox balance, as well as in neurodegenerative and neurological disorders (Baxter and Hardingham, 2016; Bell and Hardingham, 2011a, 2011b; Paoletti et al., 2013). Most NMDARs contain two obligate GluN1 subunits and two GluN2 subunits, of which there are four types (2A–D), with GluN2A and GluN2B being predominant in the forebrain. The importance of the identity of NMDAR subtypes (GluN2A versus GluN2B) in humans is illustrated by the phenotypes of patients with mutations in the genes that encode them (*GRIN2A* and *GRIN2B*), which include intellectual disability, autism, epilepsy, and schizophrenia (Burnashev and Szepetowski, 2015; Hardingham and Do, 2016). The GluN2 subtype dictates NMDAR biophysical properties, including agonist affinity, open probability, and deactivation kinetics (Paoletti et al., 2013; Wyllie et al., 2013), thus influencing the downstream consequences of NMDAR activation, such as the properties of plasticity and metaplasticity in the cortex (Cooke and Bear, 2013; Philpot et al., 2003). In addition, NMDAR composition can influence downstream NMDAR signaling to synaptic plasticity, cognition, and excitotoxicity by virtue of their highly divergent cytoplasmic C-terminal domains (CTDs) (Martel et al., 2012; Ryan et al., 2008, 2013). During forebrain development, there is a shift in NMDAR subunit composition, from near-exclusively GluN2B-containing NMDARs to NMDARs containing a significant GluN2A contribution, e.g., $GluN1_2$-$GluN2A_2$ diheteromeric receptors and $GluN1_2$-GluN2A-GluN2B triheteromeric receptors (Wyllie et al., 2013). This has been referred to as the "switch" in composition, which is partly experience dependent and takes place over postnatal weeks 2–5 in mice.

A prominent model for the mechanism by which GluN2A becomes incorporated into NMDARs at the expense of GluN2B involves a series of phosphorylation events centered on the CTD of GluN2B (CTD^{2B}) and initiated by CaMKIIα binding to its

This is an open access article under the CC BY license (http://creativecommons.org/licenses/by/4.0/).

GluN2B-specific interaction site (Lussier et al., 2015; Sanz-Clemente et al., 2010, 2013). CaMKIIα has been proposed to recruit casein kinase 2 (CK2) to phosphorylate CTD^{2B} at serine-1480, leading to the dissociation of the MAGUK-Fyn complex and reduction in CTD^{2B} tyrosine-1472 phosphorylation (a target of Fyn), destabilizing GluN2B's presence at the synapse and ultimately triggering AP-2-mediated endocytosis (Lavezzari et al., 2003; Sanz-Clemente et al., 2010, 2013). Because this model is based on observations of the properties of ectopically expressed mutant subunits, we wanted to gain insight into the role of GluN2B CTD-specific sequences in the developmental switch of endogenous NMDAR subunits and have approached this by studying receptors that have been modified at the genomic level.

RESULTS

Mutation of the GluN2B CaMKII Site Influences Phosphorylation of Its CTD

To study the role of the endogenous CTD^{2B} CaMKII binding site, we generated a knockin mouse, containing three mutations in the GluN2B CTD^{2B} sub-region (L1298A/R1300N/S1303D), hereafter referred to as GluN2B$^{\Delta CaMKII}$ (Figures 1A and S1A). The combined L1298A/R1300N mutation is sufficient to abrogate CaMKII binding to GluN2B, as has the additional S1303D mutation *in vitro* (Strack et al., 2000), which mimics the CaMKII-dependent phosphorylation event that inhibits its own binding (Strack et al., 2000). The homozygote GluN2B$^{\Delta CaMKII/\Delta CaMKII}$ mice were healthy and fertile, but to provide wild-type controls for the GluN2B$^{\Delta CaMKII/\Delta CaMKII}$ neurons within the same litter, our approach was to perform heterozygous intercrosses. Co-immunoprecipitation of NMDARs (using an N-terminal antibody to GluN2B) with CaMKIIα was reduced by around 40% (Figures 1H and 1I), a reduction similar to that observed with a previously described GluN2B L1298A/R1300N knockin (Halt et al., 2012). The remaining co-immunoprecipitation may be due to CaMKIIα association with GluN1 (Leonard et al., 2002).

We first studied CTD^{2B} phosphorylation in GluN2B$^{\Delta CaMKII/\Delta CaMKII}$ neurons, observing a reduction in CTD^{2B} Ser-1480 phosphorylation and an increase in Tyr-1472 phosphorylation in cortical neurons at both day in vitro 9 (DIV9) and DIV16 (Figures 1B and 1C), consistent with a role for CaMKII in recruiting casein kinase 2 to CTD^{2B}, causing a reduction in Fyn recruitment (Lavezzari et al., 2003; Sanz-Clemente et al., 2010, 2013). As expected, GluN2B$^{\Delta CaMKII/\Delta CaMKII}$ was also immunonegative for the phospho-Ser-1303 site (Figure 1C).

It has been shown that overexpressing constitutively active Fyn enhances synaptic localization of wild-type GluN2B by around 15% in a Tyr-1472-dependent manner (Prybylowski et al., 2005), so we studied synaptic versus extrasynaptic localization. At an age (DIV9) when NMDAR currents are almost entirely mediated by GluN2B-containing NMDARs, GluN2B$^{\Delta CaMKII/\Delta CaMKII}$ neurons were found to have a similar proportion of extrasynaptic NMDAR currents as neurons from GluN2B$^{WT/WT}$ littermates (Figure 1E). We also measured synaptic and extrasynaptic GluN2B levels in the neocortex (Figure S1B) and the hippocampus (Figure S1C) of P28 GluN2B$^{\Delta CaMKII/\Delta CaMKII}$ mice, using biochemical fractionation of synaptosome preparations (Martel et al., 2012), again revealing no genotype-dependent differences. Thus, the relatively modest changes in CTD phosphorylation in GluN2B$^{\Delta CaMKII/\Delta CaMKII}$ neurons appeared to be insufficient to significantly affect NMDAR localization. This is consistent with observations that complete exchange of the GluN2B CTD with that of GluN2A does not influence synaptic versus extrasynaptic localization (Martel et al., 2012).

The GluN2B CaMKII Site Is Dispensable for the Developmental Switch *In Vitro* and *In Vivo*

To investigate the role of the endogenous CTD^{2B} CaMKII in the developmental change in NMDAR subunit composition, we first studied primary cortical neurons, which show increased GluN2A incorporation during the 2nd week *in vitro*. NMDAR current density in the cortical neurons increased between DIV8 and DIV14–18 and was not different in GluN2B$^{\Delta CaMKII/\Delta CaMKII}$ neurons versus GluN2B$^{WT/WT}$ neurons (Figure 1D). Moreover, we observed the expected developmental increase in the GluN2A:GluN2B ratio (protein) in both genotypes (Figures 1F and 1G). To assess changes in subunit composition, a standard approach is to assess the sensitivity of NMDAR currents to a GluN2B-selective antagonist, such as ifenprodil. We observed a drop in the ifenprodil sensitivity of NMDAR currents between DIV8 and DIV14–18 in GluN2B$^{WT/WT}$ neurons as expected (Figure 1J) but also a similar drop in currents from GluN2B$^{\Delta CaMKII/\Delta CaMKII}$ neurons.

The degree of the drop in ifenprodil sensitivity is limited by the fact that GluN1$_2$-GluN2B$_2$ diheteromeric receptors are only maximally blocked by around 80%, and GluN1$_2$-GluN2A-GluN2B triheteromeric receptors, which contribute to NMDAR currents in mature forebrain neurons, are still blocked by around 30% (Stroebel et al., 2014). We therefore took an additional approach, exploiting the strong GluN2B-specific potentiation of NMDAR currents by spermine at -30 mV (pH 6.5; Mony et al., 2011), observed in GluN1$_2$-GluN2B$_2$ NMDARs, but not GluN1$_2$-GluN2A-GluN2B or GluN1$_2$-GluN2A$_2$ NMDARs (Stroebel et al., 2014). We validated the approach in neurons from a GluN2A$^{-/-}$ rat line we recently generated: cortical neurons from GluN2A$^{+/+}$, but not GluN2A$^{-/-}$, littermates showed a developmental drop in spermine potentiation of NMDAR currents (Figure S1D). Applying this technique to GluN2B$^{WT/WT}$ versus GluN2B$^{\Delta CaMKII/\Delta CaMKII}$ mouse cortical neurons, we observed a developmental drop in spermine potentiation in GluN2B$^{WT/WT}$ neurons, which was equally strong in GluN2B$^{\Delta CaMKII/\Delta CaMKII}$ neurons (Figures 1K and 1L). Collectively, these data show that the GluN2B CaMKII site is not required for normal developmental changes in NMDAR subunit composition in primary cortical neurons.

An advantage of employing a knockin mouse model is that the role of the GluN2B CaMKII site can be studied *in vivo*. In protein extracts from the neocortex and hippocampus of GluN2B$^{WT/WT}$ and GluN2B$^{\Delta CaMKII/\Delta CaMKII}$ mice, we observed the expected increase in GluN2A expression between postnatal day 14 (P14) and P28 (Figures 1M, 1N, and S1E). We prepared postsynaptic density (PSD) fractions and measured the GluN2A:GluN2B ratio, finding that the expected developmental increase in the GluN2A:GluN2B ratio in synaptic NMDARs in GluN2B$^{WT/WT}$ mice was also observed in GluN2B$^{\Delta CaMKII/\Delta CaMKII}$ mice in the

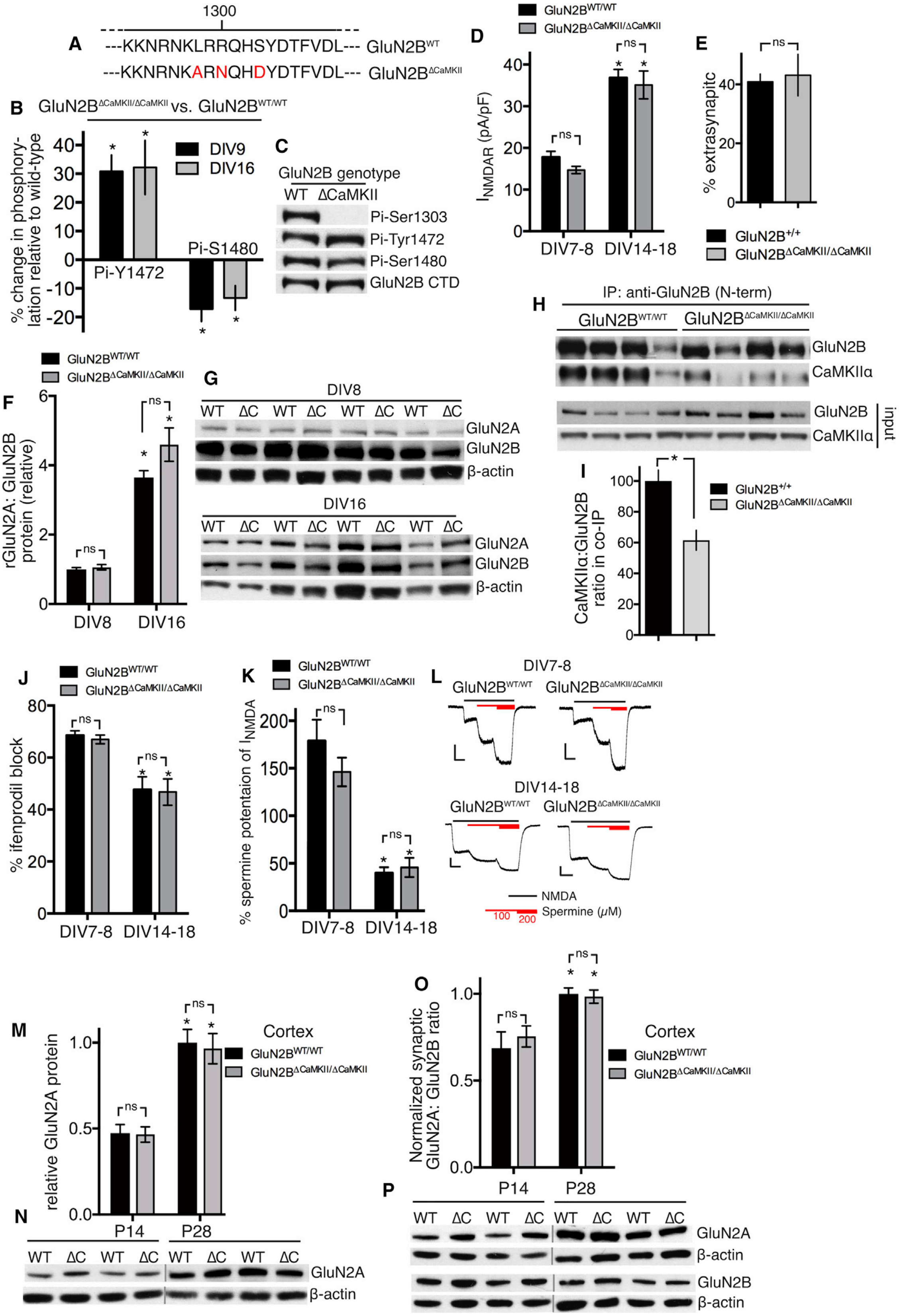
A
1300
---KKNRNKLRRQHSYDTFVDL--- GluN2BWT
---KKNRNKARNQHDYDTFVDL--- GluN2BΔCaMKII
B
GluN2BΔCaMKII/ΔCaMKII vs. GluN2BWT/WT
% change in phosphorylation relative to wild-type
DIV9
DIV16
Pi-Y1472
Pi-S1480
C
GluN2B genotype
WT ΔCaMKII
Pi-Ser1303
Pi-Tyr1472
Pi-Ser1480
GluN2B CTD
D
GluN2BWT/WT
GluN2BΔCaMKII/ΔCaMKII
INMDAR (pA/pF)
DIV7-8 DIV14-18
E
% extrasynapitc
GluN2B+/+
GluN2BΔCaMKII/ΔCaMKII
F
rGluN2A: GluN2B protein (relative)
DIV8 DIV16
G
DIV8
WT ΔC WT ΔC WT ΔC WT ΔC
GluN2A
GluN2B
β-actin
DIV16
H
IP: anti-GluN2B (N-term)
GluN2BWT/WT GluN2BΔCaMKII/ΔCaMKII
GluN2B
CaMKIIα
input
I
CaMKIIα:GluN2B ratio in co-IP
J
% ifenprodil block
K
% spermine potentaion of INMDA
L
DIV7-8
DIV14-18
NMDA
Spermine (μM)
100 200
M
relative GluN2A protein
Cortex
P14 P28
N
O
Normalized synaptic GluN2A: GluN2B ratio
P

neocortex (Figures 1O and 1P) and hippocampus (Figure S1F). Thus, targeted mutation of the endogenous GluN2B CaMKII site has no apparent effect on the developmental shift in synaptic NMDAR subunit composition.

The GluN2B CaMKII Site Is Dispensable for Synaptogenesis and Theta Burst LTP

The GluN2B CaMKII site has been proposed to be important for synaptic NMDAR signaling to the ERK1/2 pathway: overexpressing a CaMKIIα mutant defective in GluN2B binding reduces NMDAR-dependent ERK1/2 activation (El Gaamouch et al., 2012). We analyzed basal ERK1/2 activity in DIV9 cortical cultures, which is maintained by synaptic NMDAR activity (sensitive to TTX and MK-801) and is partly CaM-kinase-dependent (sensitive to KN-62; Chandler et al., 2001; El Gaamouch et al., 2012). Studying wild-type, GluN2B$^{\Delta CaMKII/\Delta CaMKII}$, and GluN2B$^{2A(CTR)/2A(CTR)}$ neurons, we found a significant effects of both TTX, MK-801, and KN-62 on basal levels, confirming previous observations, but no genotype-dependent differences in ERK1/2 activity (Figures 2A and 2B). To test whether any GluN2B-specific CTD sequences are needed for signaling to ERK1/2, we performed the same experiment on neurons cultured from GluN2B$^{2A(CTR)/2A(CTR)}$ pups, a knockin line in which GluN2B CTD has been replaced by that of GluN2A (Martel et al., 2012; Ryan et al., 2013). Compared to neurons cultured from GluN2B$^{WT/WT}$ littermates, we again observed no genotype-dependent differences in activity-dependent ERK1/2 activity (Figures 2A and 2C), suggesting that this does not have a requirement for GluN2B-specific CTD sequences.

An additional putative role of the GluN2B CaMKII interaction site is in excitatory synaptogenesis, based on the effects of overexpression of a GluN2B mutant impaired in CaMKII binding (RS/QD: R1300Q;S1303D; Gambrill and Barria, 2011). Analysis of miniature excitatory postsynaptic current (mEPSC) size and frequency revealed that they were unaltered in GluN2B$^{\Delta CaMKII/\Delta CaMKII}$ mice (Figures 2D–2F). Consistent with this, field excitatory postsynaptic potential (fEPSP) recordings by multi-electrode array revealed similar input-output curves in both genotypes (Figure S2A). Also, a comparison of fEPSP slopes and presynaptic fiber volley amplitudes in fEPSP recordings by conventional glass electrodes also revealed no difference between genotypes (Figures S2B and S2C). Given that CaMKIIα activity is implicated in synaptogenesis, we looked at the levels of phospho-Thr286 CaMKIIα in hippocampal postsynaptic densities and found them to be similar in mice of both genotypes also (Figure S2D). We also probed the NMDAR properties and subunit composition in the hippocampal CA1 synapses. NMDAR:AMPAR ratios were similar in neurons of GluN2B$^{WT/WT}$ and GluN2B$^{\Delta CaMKII/\Delta CaMKII}$ mice (Figure 2G), as was the decay kinetics of EPSCs recorded at +40 mV (Figures 2H and 2I), which is dictated by the dissociation rate of glutamate from NMDARs and is slower for GluN2B than GluN2A (Paoletti et al., 2013).

An additional putative role of the GluN2B CaMKII interaction site is in hippocampal theta burst (TBS) long-term potentiation (Barria and Malinow, 2005), known to involve both CaMKII and ERK1/2 activation (Winder et al., 1999; Barria and Malinow, 2005; Ito et al., 1991). We found that TBS-long term potentiation (LTP) was not significantly different in GluN2B$^{\Delta CaMKII/\Delta CaMKII}$ versus GluN2B$^{WT/WT}$ slices (Figures 2J and 2K; n = 8 animals per genotype), suggesting that the GluN2B CaMKII site is not critical for this form of NMDAR-dependent synaptic plasticity.

Distinct GluN2 CTDs Are Not Required for the NMDAR Developmental Switch

We next addressed a more general question as to whether the NMDAR subunit switch has any requirement for GluN2-specific CTD sequences. To investigate this, we utilized another knockin mouse, GluN2A$^{2B(CTR)}$ (Figure 3A), in which we replaced the exon encoding the CTD of GluN2A with that of GluN2B (Ryan et al., 2013), meaning that both GluN2A and GluN2B have the same CTD. NMDAR current density in GluN2A$^{2B(CTR)/2B(CTR)}$ neurons

Figure 1. The GluN2B CaMKII Site Is Dispensable for the Developmental Switch *In Vitro*

(A) Schematic of the GluN2B$^{\Delta CaMKII}$ amino acid changes.

(B and C) Altered GluN2B phosphorylation in GluN2B$^{\Delta CaMKII/\Delta CaMKII}$ neurons. Cortical neuronal extracts were prepared and analyzed by western blot with the indicated antibodies, normalized to total GluN2B. (B) shows quantitation (mean ± SEM here and throughout), and (C) shows an example. *$p < 0.05$ versus GluN2B$^{+/+}$ (n = 4).

(D) NMDAR current density at the indicated ages for GluN2B$^{WT/WT}$ and GluN2B$^{\Delta CaMKII/\Delta CaMKII}$ neurons. *$p < 0.05$ versus DIV7 or DIV8 of the same genotype; two-way ANOVA plus Sidak's post hoc test (GluN2B$^{WT/WT}$: n = 14 [DIV7 or DIV8], n = 18 [DIV14–18]; GluN2B$^{\Delta CaMKII/\Delta CaMKII}$: n = 15 [DIV7 or DIV8], n = 17 [DIV14–18]).

(E) Extrasynaptic NMDAR currents were measured in GluN2B$^{WT/WT}$ and GluN2B$^{\Delta CaMKII/\Delta CaMKII}$ neurons (n = 8 per genotype).

(F and G) GluN2A and GluN2B expression analyzed by western blot at DIV8 and DIV16 for GluN2B$^{WT/WT}$ and GluN2B$^{\Delta CaMKII/\Delta CaMKII}$ neurons. (F) shows quantitation and (G) shows an example. *$p < 0.05$ versus DIV8 of the same genotype; two-way ANOVA plus Sidak's post hoc test (n = 8).

(H and I) Neocortices of GluN2B$^{WT/WT}$ and GluN2B$^{\Delta CaMKII/\Delta CaMKII}$ mice were subjected to immunoprecipitation with an antibody to the N terminus of GluN2B, followed by analysis of GluN2B and CaMKIIα content. (H) shows an example and (I) shows quantitation. *$p < 0.05$; n = 8 per genotype.

(J) Percentage blockade of NMDAR currents by ifenprodil (3 μM) at the indicated stages for GluN2B$^{WT/WT}$ and GluN2B$^{\Delta CaMKII/\Delta CaMKII}$ neurons. *$p < 0.05$ versus DIV7 or DIV8 of the same genotype; two-way ANOVA plus Sidak's post hoc test (GluN2B$^{WT/WT}$: n = 14 [DIV7 or DIV8], n = 18 [DIV14–18]; GluN2B$^{\Delta CaMKII/\Delta CaMKII}$: n = 15 [DIV7 or DIV8], n = 17 [DIV14–18]).

(K and L) Percentage potentiation of NMDAR currents by 100 μM spermine was measured at the indicated stages for GluN2B$^{WT/WT}$ and GluN2B$^{\Delta CaMKII/\Delta CaMKII}$ neurons. *$p < 0.05$ versus DIV7 or DIV8 of the same genotype; two-way ANOVA plus Sidak's post hoc test (GluN2B$^{WT/WT}$: n = 13 [DIV7 or DIV8], n = 18 [DIV14–18]; GluN2B$^{\Delta CaMKII/\Delta CaMKII}$: n = 18 [DIV7 or DIV8], n = 14 [DIV14–18]). (K) shows quantitation and (L) shows example traces; scale bar: 200 pA/2 s.

(M and N) Neocortical extracts from P14 and P28 mice were analyzed for GluN2A expression, normalized to β-actin. (M) shows quantitation and (N) shows an example. *$p < 0.05$ versus P14 of the same genotype; two-way ANOVA plus Sidak's post hoc test (n = 8).

(O and P) Post-synaptic density (PSD) extracts from P14 and P28 mice of the indicated genotype were analyzed for GluN2A and GluN2B expression, normalized to β-actin, and the ratio calculated. (O) shows quantitation and (P) shows an example. *$p < 0.05$ versus P14 of the same genotype; two-way ANOVA plus Sidak's post hoc test (n = 8).

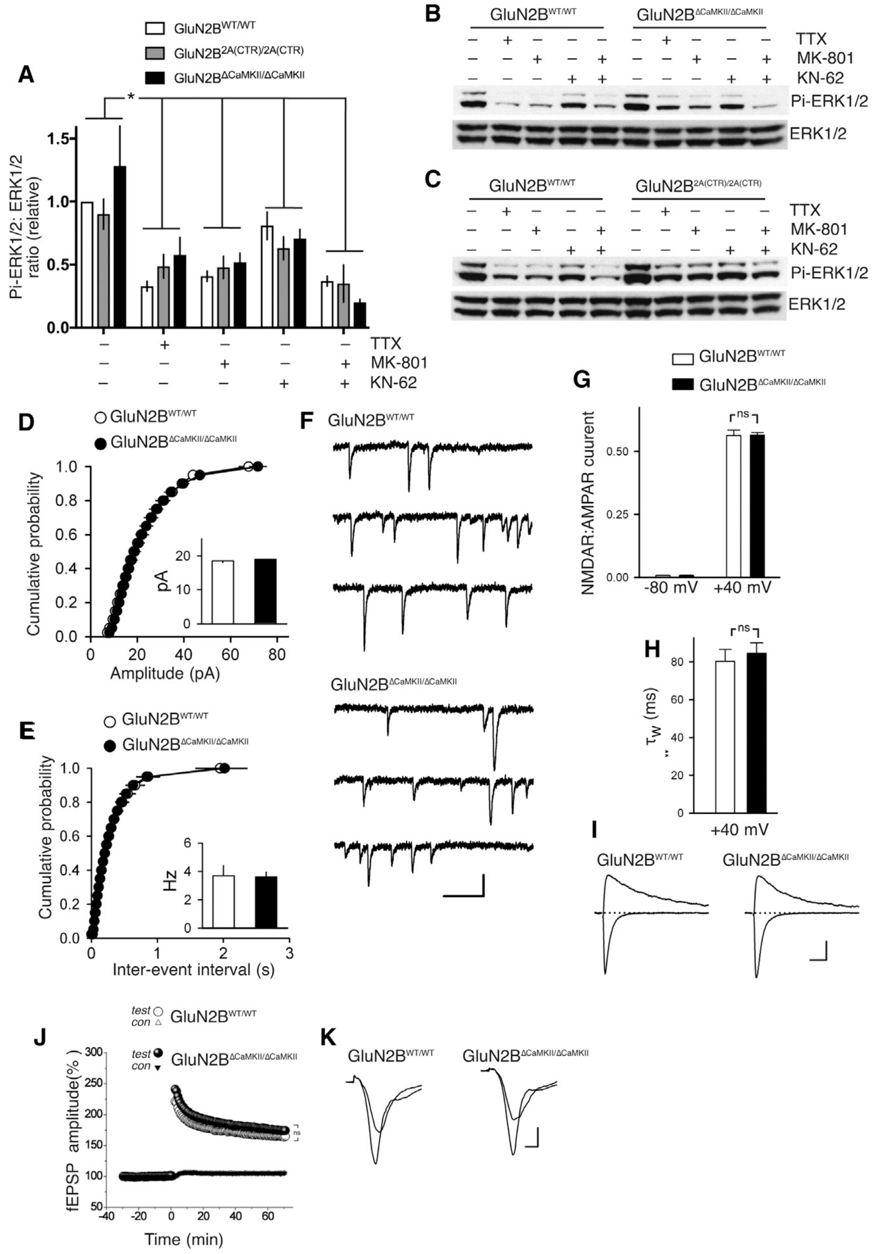

Figure 2. The GluN2B CaMKII Binding Site Is Dispensable for Theta Burst LTP

(A–C) Activity-dependent signaling to ERK1/2 does not require GluN2B CTD-specific sequences. DIV9 cortical neurons of the indicated genotypes were treated with TTX (500 nM), KN-62 (10 μM), or MK-801 (10 μM) for 1 hr, after which protein extracts were made and subjected to western blot analysis for phospho-ERK1/2 levels, normalized to total ERK1/2. (A) shows quantitation and (B) and (C) show example blots.*$p < 0.05$ two-way ANOVA plus Dunnett's post hoc test. No genotype-dependent effects were observed ($p = 0.39$) or genotype-drug interactions ($p = 0.16$; n = 8 GluN2B$^{WT/WT}$; n = 4 GluN2B$^{\Delta CaMKII/\Delta CaMKII}$; n = 4 GluN2B$^{2A(CTR)/2A(CTR)}$).

(D–F) Cumulative probability plots show amplitude (D) and frequency (E) of miniature EPSCs in GluN2B$^{WT/WT}$ and GluN2B$^{\Delta CaMKII/\Delta CaMKII}$ slices. Results are from 11 cells from 3 GluN2B$^{WT/WT}$ mice and 13 cells from 3 GluN2B$^{\Delta CaMKII/\Delta CaMKII}$ mice; $p = 0.75$ (amplitude); $p = 0.94$ (frequency); unpaired t test. (F) shows example traces. Scale bar: 10 pA/100 ms.

(G) NMDAR:AMPAR EPSC ratios at −80 and +40 mV was measured in GluN2B$^{WT/WT}$ (n = 25; 4 mice) and GluN2B$^{\Delta CaMKII/\Delta CaMKII}$ (n = 30; 4 mice) neurons (unpaired t test; $p = 0.95$).

(H) Weighted time constant (τw) for the decay of EPSCs recorded in (A) at +40 mV was determined using double exponential fits (unpaired t test; $p = 0.63$).

(I) Example traces. Scale bar: 100 pA/20 ms.

(J and K) Theta-burst stimulation elicited pathway-specific LTP of synaptic transmission in hippocampal CA1 area. Normalized magnitude of this potentiation 60–65 min after LTP induction did not differ significantly in GluN2B$^{WT/WT}$ mice (29 slices; n = 9) compared to GluN2B$^{\Delta CaMKII/\Delta CaMKII}$ (35 slices; n = 11); $p = 0.162$ (two-way nested ANOVA). (J) shows quantitation of data and (K) shows example traces before and after LTP induction.

(K) Traces show example fEPSP traces immediately before and 1 hr after theta-burst stimulation. Scale bar: 0.5 mV/2 ms.

was found to be no different to GluN2A$^{WT/WT}$ neurons (Figure 3B). Importantly, the developmental drop in NMDAR current potentiation by spermine was identical in GluN2A$^{2B(CTR)/2B(CTR)}$ and GluN2A$^{WT/WT}$ neurons (Figures 3C and 3D), as was ifenprodil sensitivity of NMDAR currents (Figure S3A). We also observed no difference in the synaptic versus extrasynaptic localization of NMDARs: in DIV14–18 neurons, neither the proportion of extrasynaptic NMDAR currents nor their sensitivity to ifenprodil was different in GluN2A$^{2B(CTR)/2B(CTR)}$ neurons compared to GluN2A$^{WT/WT}$ neurons (Figures S3B and S3C). Moreover, the developmental increase in GluN2A expression in the neocortex and hippocampus of GluN2A$^{2B(CTR)/2B(CTR)}$ and GluN2B$^{WT/WT}$ mice was indistinguishable (Figures 3E, 3F, and S3D). Furthermore, the developmental increase in the synaptic GluN2A:GluN2B ratio in cortical (Figures 3G and 3H) and hippocampal (Figure S3E) tissue was also similar. Thus, even when GluN2A and GluN2B possess identical CTDs, the developmental shift in NMDAR subunit composition, and their targeting to synaptic or extrasynaptic locations, proceeds normally *in vitro* and *in vivo*.

Increased Expression of GluN2A Is a Key Determinant of the Shift in Subunit Composition

It has been proposed that epigenomic repression of GluN2B is a contributing factor to the developmental increase in GluN2A:GluN2B ratio in the hippocampus (Rodenas-Ruano et al., 2012). However, the extent to which GluN2B repression drives the subunit switch in the cortex, compared to GluN2A

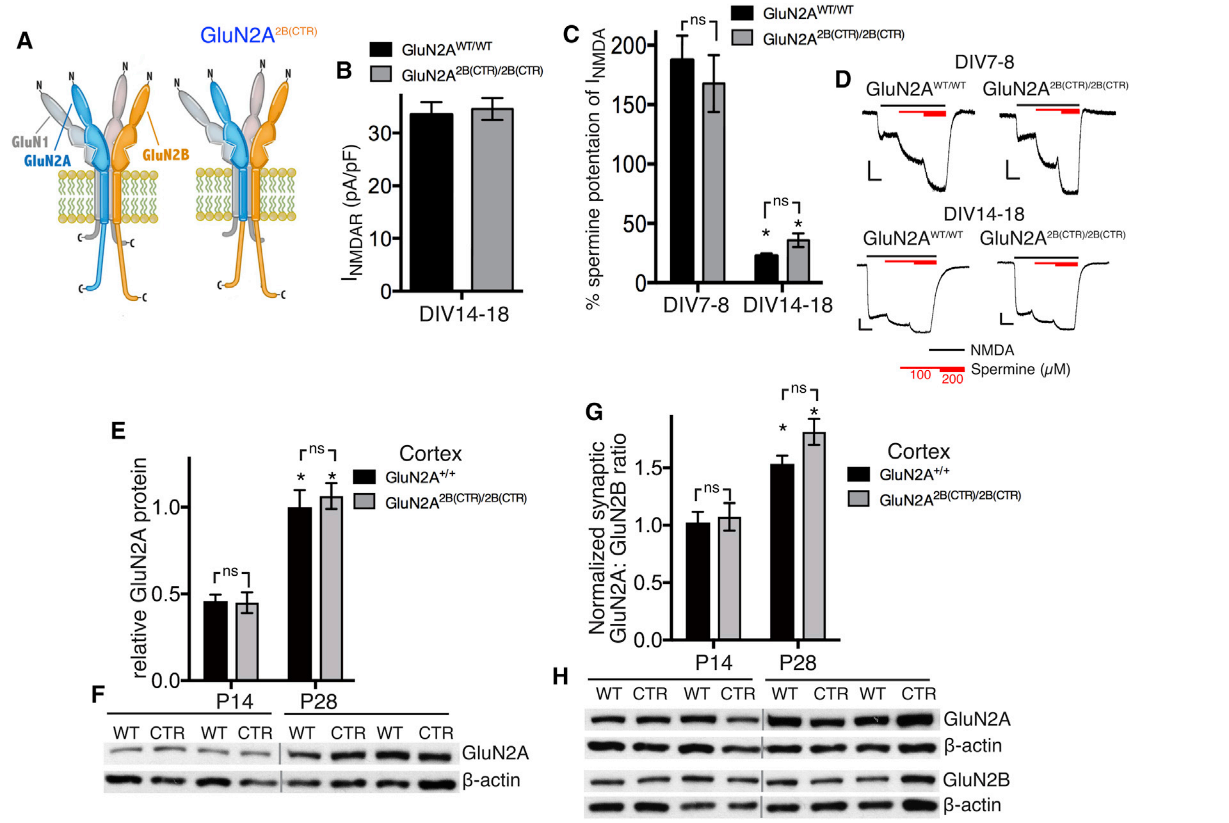

Figure 3. Distinct GluN2 CTDs Are Not Required for the 2B-to-2A Switch

(A) Schematic illustrating the C-terminal domain exchange in the GluN2A$^{2B(CTR)/2B(CTR)}$ mouse.

(B) NMDAR current density in GluN2A$^{WT/WT}$ (n = 30) and GluN2A$^{2B(CTR)/2B(CTR)}$ (n = 24) neurons, recorded DIV14–18.

(C and D) Percentage potentiation of NMDAR currents by 100 μM spermine was measured in GluN2A$^{WT/WT}$ and GluN2A$^{2B(CTR)/2B(CTR)}$ neurons. *$p < 0.05$ versus DIV7 or DIV8 of the same genotype; two-way ANOVA plus Sidak's post hoc test (GluN2A$^{WT/WT}$: n = 19 [DIV7 or DIV8], n = 18 [DIV14–18]; GluN2A$^{2B(CTR)/2B(CTR)}$: n = 16 [DIV7 or DIV8], n = 17 [DIV14–18]). (C) shows quantitation of data and (D) shows example traces; scale bar: 200 pA/2 s.

(E and F) Neocortical extracts from P14 and P28 mice of the indicated genotype were analyzed for GluN2A expression, normalized to β-actin. *$p < 0.05$ versus P14 of the same genotype; two-way ANOVA plus Sidak's post hoc test (n = 8). (E) shows quantitation and (F) shows example blots.

(G and H) PSD extracts from P14 and P28 mice of the indicated genotype were analyzed for GluN2A and GluN2B expression, normalized to β-actin, and the ratio calculated and scaled such that the ratio at P14 for WT = 1. *$p < 0.05$ versus P14 of the same genotype; two-way ANOVA plus Sidak's post hoc test (n = 8). (G) shows quantitation and (H) shows an example blot.

upregulation, is unclear. We recently showed that GluN2B protein levels are around 10-fold higher than GluN2A in the adult cortex (Frank et al., 2016), suggesting that the GluN2A:GluN2B ratio is more sensitive to an increase in GluN2A than an equivalent fall in GluN2B. We measured total GluN2B expression in the neocortex at P14 and P28 from wild-type, GluN2A$^{2B(CTR)/2B(CTR)}$, and GluN2B$^{\Delta CaMKII/\Delta CaMKII}$ mice and found no evidence for decreasing GluN2B expression (Figures 4A–4D), consistent with other studies on the cortex (Liu et al., 2004; Sheng et al., 1994). Thus, the developmental shift in NMDAR subunit composition in the developing cortex can commence without the substantial repression of GluN2B expression, as well as without GluN2B CTD-dependent removal of GluN2B-containing NMDARs from the synapse (Figure 3). This suggests that the overriding factor is simply the increasing level of GluN2A expression. Indeed, by analyzing spermine potentiation of currents in DIV14 and DIV15 cortical neurons from GluN2A$^{+/+}$, GluN2A$^{+/-}$, and GluN2A$^{-/-}$ rats, we observed a negative correlation with gene copy number (Figures 4H and 4I). This points to impairment in the replacement of GluN1$_2$-GluN2B$_2$ NMDARs with GluN1$_2$-GluN2A-GluN2B and GluN1$_2$-GluN2A$_2$ NMDARs, due to haploinsufficiency of the *Grin2a* gene, suggesting that GluN2A levels are the limiting factor in dictating the switch. As expected, the reverse was also found: GluN2A overexpression in young rat or mouse neurons has a very modest effect on NMDAR currents (Martel et al., 2012) but resulted in a very strong reduction of NMDAR current potentiation by spermine (Figures 4E–4G). Thus, a major determinant of the developmental shift in NMDAR subunit composition is the increasing expression of GluN2A.

DISCUSSION

Probing Channel Domain Function Using Knockin Mouse Models

Our experiments point to the value of probing putative signaling domains within ion channels and receptors by targeted genetic

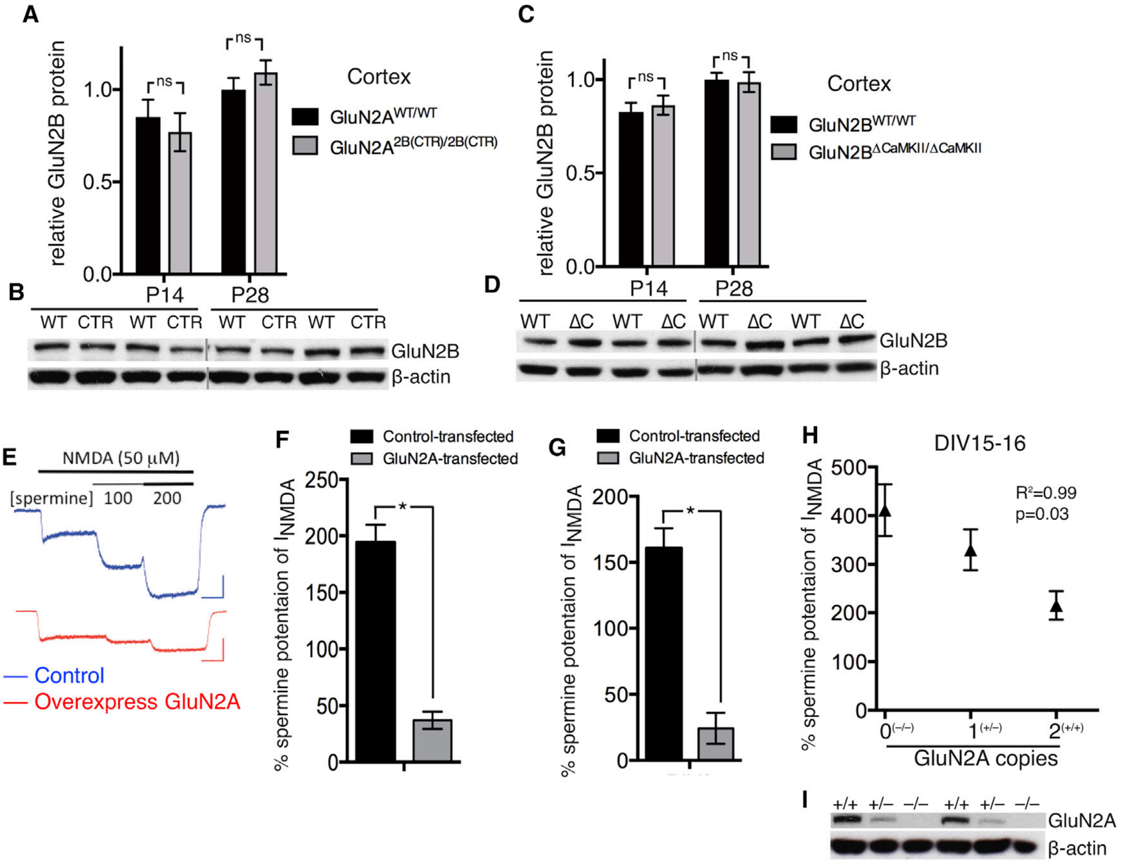

Figure 4. GluN2A Expression Is Sufficient to Displace GluN2B from NMDARs

(A–D) Total GluN2B expression was measured in extracts from the neocortex at the indicated stages for the indicated genotypes (n = 8 per genotype). (A) and (B) show quantitation and example blots, respectively, for GluN2A$^{WT/WT}$ versus GluN2A$^{2B(CTR)/2B(CTR)}$. (C) and (D) show quantitation and example blots, respectively, for GluN2A$^{WT/WT}$ vs. GluN2B$^{\Delta CaMKII/\Delta CaMKII}$.

(E–G) Ectopic GluN2A expression is sufficient to displace GluN2B from NMDARs. Young mouse (E and F) and rat (G) neurons at DIV7 were transfected with a control (β-globin) or GluN2A-encoding vector and spermine (100 μM) potentiation of NMDAR currents measured 72 hr later. *p < 0.05 (F: n = 10 of both condition; G: n = 8 control; n = 10 GluN2A).

(H) Percentage potentiation of NMDAR currents by spermine (200 μM) in DIV15 or DIV16 rat cortical neurons was measured at the indicated stages for GluN2A$^{+/+}$, GluN2A$^{+/-}$, and GluN2A$^{-/-}$ genotypes. Person r correlation coefficient: −0.9954; p = 0.031 (one-tailed test); n = 12 cells per genotype.

(I) Western blot illustrating GluN2A expression in GluN2A$^{+/+}$, GluN2A$^{+/-}$, and GluN2A$^{-/-}$ neurons.

(I) A western blot confirming the absence of GluN2A expression in GluN2A$^{-/-}$ and an intermediate expression level in GluN2A$^{+/-}$ neurons.

approaches, as opposed to reliance on ectopic expression of mutant receptors. For example, compared to overexpression of wild-type GluN2B, overexpression of the GluN2B CaMKII site R1300Q/S1303D mutant in cultured hippocampal neurons impairs functional synaptogenesis, assayed by mEPSC frequency and spine number (Gambrill and Barria, 2011). In contrast, we found normal mEPSC size and frequency in hippocampal slices of GluN2B$^{\Delta CaMKII/\Delta CaMKII}$ mice, consistent with a report of normal synaptogenesis in an independently created CaMKII site mutant knockin mouse (Halt et al., 2012). Furthermore, a recent study proposed that GluN2B's CamKIIα interaction site is important for correct basal AMPA-receptor-mediated synaptic transmission (Incontro et al., 2018), based on results of acute virus-delivered CRISPR deletion of the endogenous GluN2B gene in slice culture and ectopic expression of mutant versus wild-type (WT) subunits. In contrast, we see normal basal hippocampal synaptic transmission in GluN2B$^{\Delta CaMKII/\Delta CaMKII}$ mice (Figures 2 and S2), again consistent with the observations made previously in a different knockin model (Halt et al., 2012). Furthermore, overexpression of another GluN2B CaMKII site mutant (S1303E) led to a higher increase in GluN2B surface expression than WT GluN2B overexpression (Sanz-Clemente et al., 2013), whereas we found normal NMDAR currents in GluN2B$^{\Delta CaMKII/\Delta CaMKII}$ neurons. Moreover, expression of the CaMKII site mutant of GluN2B increased the GluN2B:GluN2A ratio at functional NMDARs in slice cultures, compared to the wild-type GluN2B, as measured by studying the EPSC decay constant (Sanz-Clemente et al., 2013). In contrast, our recordings in acute hippocampal slices revealed unaltered EPSC decay constants in GluN2B$^{\Delta CaMKII/\Delta CaMKII}$ mice, indicative of a normal synaptic GluN2B:GluN2A ratio, confirmed by biochemical fractionation. What could explain the discrepancy between

observations based on knockin mouse lines versus overexpression of mutant subunits? One difference is that the latter may alter the relative stoichiometry of GluN2B and CaMKIIα, which could influence CaMKIIα interactions in the PSD. In the rat brain PSD, CaMKIIα is 50 times more abundant than GluN2B and 35- to 100-fold more abundant than GluN1 (Lowenthal et al., 2015; Cheng et al., 2006). As such, mutating the endogenous GluN2B CaMKIIα site in a knockin is unlikely to influence the total synaptic or PSD-localized CaMKIIα. However, overexpression of GluN2B and mutant variants could alter the stoichiometric balance and cause the interaction of GluN2B with a greater proportion of CaMKIIα at the synapse and elsewhere. Of note, however, we did find that germline mutation of the GluN2B CaMKII site rendered neurons less vulnerable to excitotoxic insults (unpublished observation), consistent with previous findings based on overexpression of mutant subunits lacking this site (Vieira et al., 2016). Thus, results from ectopic subunit expression are not always in disagreement with conclusions derived from equivalent knockin models.

Although the GluN2B CaMKII site may have a limited role in the general developmental subunit switch in the cortex, it remains a possibility that it plays a role in more specialized situations, such as the experience or activity-dependent element of the 2B-to-2A switch that occurs in the developing visual cortex (Quinlan et al., 1999). It will be of interest to determine whether this process has a requirement for GluN2 CTD subtype-specific events, particularly because overexpression studies have implicated the GluN2B CaMKII site in activity-dependent subunit switching (Sanz-Clemente et al., 2013).

Additionally, although our study argues against a role for the GluN2B CaMKII site-dependent recruitment of CK2 in the NMDAR subunit switch, it remains possible that CK2 could still play a role in phosphorylating GluN2B Ser-1480, disrupting its interactions with MAGUKs, leading to reduced surface expression (Chung et al., 2004) independent of the GluN2B CaMKII site. We considered investigating the influence of the CK2 inhibitor TBB, used previously to implicate CK2 in the switch. However, TBB is known to inhibit kinases DYRK1A, DYRK2, DYRK3, PIM2, PIM3, and HIPK2 just as effectively as it inhibits CK2 (Pagano et al., 2008). Indeed, the TBB target DYRK1A has been shown to phosphorylate and positively influence the surface expression of GluN2A (Grau et al., 2014; Altafaj et al., 2008). Thus, DYRK1A inhibition by TBB could conceivably reduce GluN2A levels via this mechanism. Another potential issue is TBB cytotoxicity (Qaiser et al., 2014), so deletion of CK2 may be a better approach to directly probe its role in NMDAR subunit switching.

Interdependence of GluN2B Phosphorylation Sites

Our observations align with predictions based on previous studies (Sanz-Clemente et al., 2013) that mutation of the GluN2B CaMKII site would reduce Ser-1480 phosphorylation (a CK2 site; Sanz-Clemente et al., 2010) and increase Tyr-1472 phosphorylation (a Fyn site; Nakazawa et al., 2001). However, although increased Fyn-driven Tyr-1472 phosphorylation prevents AP-2-mediated GluN2B endocytosis (Prybylowski et al., 2005; Roche et al., 2001; Zhang et al., 2008), we did not observe increased synaptic GluN2B in GluN2B$^{\Delta CaMKII/\Delta CaMKII}$ neurons. One possible explanation is that the changes in phosphorylation we observe (around 30%) were too small to have a significant effect on synaptic GluN2B levels. Indeed, constitutive activation of Fyn increased synaptic GluN2B-mediated NMDAR currents by only around 15%, and a similar difference was observed under basal conditions between GluN2B and non-phosphorylatable GluN2B-Y1472A overexpressing neurons (Prybylowski et al., 2005). In a similar vein, the reduction in endocytosis observed in ectopically expressed GluN2B-Y1472A compared to ectopically expressed WT GluN2B is around 25% (Sanz-Clemente et al., 2010). Thus, the changes observed even when driving the system maximally by expressing mutant subunits and constitutively active kinases are quite modest, meaning that the relatively small changes in Y-1472 phosphorylation may not have a detectable effect on synaptic GluN2B expression.

The GluN2B CaMKII Site in Synaptic Plasticity

Our findings regarding normal excitatory synaptogenesis and synaptic transmission in the GluN2B$^{\Delta CaMKII/\Delta CaMKII}$ mouse are consistent with observations made in an independently created knockin mouse with a targeted mutation of the GluN2B CaMKII site (Halt et al., 2012). One observation where our data do not align is their reporting of a deficit (ca. 30%) in theta-burst LTP, which we did not observe. One difference in our knockin lines is our additional mutation (the phospho-mimetic S1303D), chosen because, although R1300Q and S1303D both reduce CaMKII-GluN2B interaction, the double mutant R1300Q/S1303D reduces binding even further (Strack et al., 2000). The inclusion of the S1303D in GluN2B "CaMKII site" mutants has been employed elsewhere (Barria and Malinow, 2005; Gambrill and Barria, 2011; Vieira et al., 2016) but will alter the charge at this site. However, whatever the approach used to disrupt CaMKII binding, alteration of the charge at Ser-1303 is likely to be unavoidable, because CaMKII is the major kinase at this site and forebrain neurons have substantial basal S1303 phosphorylation (McQueen et al., 2017), which will likely decrease if CaMKII association is disrupted. It is therefore likely that mutating L1298 and R1300 alone may lower S1303 phosphorylation (Halt et al., 2012), reducing the negative charge at this site, and our mutation of S1303D will increase the charge. Thus, although there is no evidence that the charge at S1303 has additional effects, this difference between the two knockin models could explain the discrepancy in the LTP experiments and requires further investigation.

Concluding Remarks

Our observations argue against a central role for CTD subtype-specific events in the normal developmental subunit shift in cortical NMDAR subunit composition and that an increase in GluN2A expression is likely the major driver of GluN2A:GluN2B ratio increases. Thus, although GluN2 subtype-specific CTD sequences do influence excitotoxicity, plasticity, protein complex formation, and behavioral repertoire (Frank et al., 2016; Martel et al., 2012; Ryan et al., 2013), their role in directing neuronal development and maturation may be less pronounced.

STAR★METHODS

Detailed methods are provided in the online version of this paper and include the following:

- KEY RESOURCES TABLE
- CONTACT FOR REAGENT AND RESOURCE SHARING
- EXPERIMENTAL MODEL AND SUBJECT DETAILS
  - Animals
  - Primary cultures
- METHOD DETAILS
  - Cell culture electrophysiological recording and analysis
  - Slice electrophysiological recordings and analysis
  - PSD fractionation, co-immunoprecipitation and western blotting
  - Transfection of cortical neurons
- QUANTIFICATION AND STATISTICAL ANALYSIS
  - Statistical analysis, equipment and settings

SUPPLEMENTAL INFORMATION

Supplemental Information includes three figures and can be found with this article online at https://doi.org/10.1016/j.celrep.2018.09.089.

ACKNOWLEDGMENTS

We thank Kathryn Elsegood, Lynsey Robertson, David Fricker, and Chih-yuan Chiang for mouse colony management and genotyping. Work in the G.E.H. lab is supported by the MRC, Wellcome Trust, Alzheimer's Research UK, and Alzheimer's Society. Work in the S.G.N.G. lab is supported by a Wellcome Technology Development Grant (202932) and an European Research Council Advanced Grant (695568).

AUTHOR CONTRIBUTIONS

Project Conceptualization, N.H.K., S.G.N.G., T.J.R., and G.E.H.; Supervision and Project Administration, N.H.K., G.E.H., S.G.N.G., T.J.O., D.J.A.W., and P.C.K.; Experimental Work, S.M., T.J.R., J.M., T.I., K.F.M.M., P.H., M.V.K., P.S.B., and M.-A.M.; Data Analysis, S.M., T.J.R., J.M., T.I., K.F.M.M., P.H., M.V.K., P.S.B., M.-A.M., T.J.O., G.E.H., and N.H.K.; Writing – Original Draft, G.E.H.; Writing – Review & Editing, all authors.

DECLARATION OF INTERESTS

The authors declare no competing interests.

Received: January 28, 2016
Revised: July 13, 2018
Accepted: September 26, 2018
Published: October 23, 2018

REFERENCES

Altafaj, X., Ortiz-Abalia, J., Fernández, M., Potier, M.C., Laffaire, J., Andreu, N., Dierssen, M., González-García, C., Ceña, V., Martí, E., and Fillat, C. (2008). Increased NR2A expression and prolonged decay of NMDA-induced calcium transient in cerebellum of TgDyrk1A mice, a mouse model of Down syndrome. Neurobiol. Dis. *32*, 377–384.

Barria, A., and Malinow, R. (2005). NMDA receptor subunit composition controls synaptic plasticity by regulating binding to CaMKII. Neuron *48*, 289–301.

Baxter, P.S., and Hardingham, G.E. (2016). Adaptive regulation of the brain's antioxidant defences by neurons and astrocytes. Free Radic. Biol. Med. *100*, 147–152.

Baxter, P.S., Martel, M.A., McMahon, A., Kind, P.C., and Hardingham, G.E. (2011). Pituitary adenylate cyclase-activating peptide induces long-lasting neuroprotection through the induction of activity-dependent signaling via the cyclic AMP response element-binding protein-regulated transcription co-activator 1. J. Neurochem. *118*, 365–378.

Baxter, P.S., Bell, K.F., Hasel, P., Kaindl, A.M., Fricker, M., Thomson, D., Cregan, S.P., Gillingwater, T.H., and Hardingham, G.E. (2015). Synaptic NMDA receptor activity is coupled to the transcriptional control of the glutathione system. Nat. Commun. *6*, 6761.

Bell, K.F., and Hardingham, G.E. (2011a). CNS peroxiredoxins and their regulation in health and disease. Antioxid. Redox Signal. *14*, 1467–1477.

Bell, K.F., and Hardingham, G.E. (2011b). The influence of synaptic activity on neuronal health. Curr. Opin. Neurobiol. *21*, 299–305.

Bell, K.F., Al-Mubarak, B., Martel, M.A., McKay, S., Wheelan, N., Hasel, P., Márkus, N.M., Baxter, P., Deighton, R.F., Serio, A., et al. (2015). Neuronal development is promoted by weakened intrinsic antioxidant defences due to epigenetic repression of Nrf2. Nat. Commun. *6*, 7066.

Burnashev, N., and Szepetowski, P. (2015). NMDA receptor subunit mutations in neurodevelopmental disorders. Curr. Opin. Pharmacol. *20*, 73–82.

Chandler, L.J., Sutton, G., Dorairaj, N.R., and Norwood, D. (2001). N-methyl D-aspartate receptor-mediated bidirectional control of extracellular signal-regulated kinase activity in cortical neuronal cultures. J. Biol. Chem. *276*, 2627–2636.

Cheng, D., Hoogenraad, C.C., Rush, J., Ramm, E., Schlager, M.A., Duong, D.M., Xu, P., Wijayawardana, S.R., Hanfelt, J., Nakagawa, T., et al. (2006). Relative and absolute quantification of postsynaptic density proteome isolated from rat forebrain and cerebellum. Mol. Cell. Proteomics *5*, 1158–1170.

Chung, H.J., Huang, Y.H., Lau, L.F., and Huganir, R.L. (2004). Regulation of the NMDA receptor complex and trafficking by activity-dependent phosphorylation of the NR2B subunit PDZ ligand. J. Neurosci. *24*, 10248–10259.

Cooke, S.F., and Bear, M.F. (2013). How the mechanisms of long-term synaptic potentiation and depression serve experience-dependent plasticity in primary visual cortex. Philos. Trans. R. Soc. Lond. B Biol. Sci. *369*, 20130284.

Delint-Ramirez, I., Fernández, E., Bayés, A., Kicsi, E., Komiyama, N.H., and Grant, S.G. (2010). In vivo composition of NMDA receptor signaling complexes differs between membrane subdomains and is modulated by PSD-95 and PSD-93. J. Neurosci. *30*, 8162–8170.

Edman, S., McKay, S., Macdonald, L.J., Samadi, M., Livesey, M.R., Hardingham, G.E., and Wyllie, D.J. (2012). TCN 201 selectively blocks GluN2A-containing NMDARs in a GluN1 co-agonist dependent but non-competitive manner. Neuropharmacology *63*, 441–449.

El Gaamouch, F., Buisson, A., Moustié, O., Lemieux, M., Labrecque, S., Bontempi, B., De Koninck, P., and Nicole, O. (2012). Interaction between αCaMKII and GluN2B controls ERK-dependent plasticity. J. Neurosci. *32*, 10767–10779.

Frank, R.A., Komiyama, N.H., Ryan, T.J., Zhu, F., O'Dell, T.J., and Grant, S.G. (2016). NMDA receptors are selectively partitioned into complexes and supercomplexes during synapse maturation. Nat. Commun. *7*, 11264.

Gambrill, A.C., and Barria, A. (2011). NMDA receptor subunit composition controls synaptogenesis and synapse stabilization. Proc. Natl. Acad. Sci. USA *108*, 5855–5860.

Grau, C., Arató, K., Fernández-Fernández, J.M., Valderrama, A., Sindreu, C., Fillat, C., Ferrer, I., de la Luna, S., and Altafaj, X. (2014). DYRK1A-mediated phosphorylation of GluN2A at Ser(1048) regulates the surface expression and channel activity of GluN1/GluN2A receptors. Front. Cell. Neurosci. *8*, 331.

Halt, A.R., Dallapiazza, R.F., Zhou, Y., Stein, I.S., Qian, H., Juntti, S., Wojcik, S., Brose, N., Silva, A.J., and Hell, J.W. (2012). CaMKII binding to GluN2B is critical during memory consolidation. EMBO J. *31*, 1203–1216.

Hardingham, G.E., and Do, K.Q. (2016). Linking early-life NMDAR hypofunction and oxidative stress in schizophrenia pathogenesis. Nat. Rev. Neurosci. *17*, 125–134.

Hardingham, N.R., Hardingham, G.E., Fox, K.D., and Jack, J.J. (2007). Presynaptic efficacy directs normalization of synaptic strength in layer 2/3 rat neocortex after paired activity. J. Neurophysiol. *97*, 2965–2975.

Hasel, P., Dando, O., Jiwaji, Z., Baxter, P., Todd, A.C., Heron, S., Márkus, N.M., McQueen, J., Hampton, D.W., Torvell, M., et al. (2017). Neurons and neuronal activity control gene expression in astrocytes to regulate their development and metabolism. Nat. Commun. *8*, 15132.

Hooper, M., Hardy, K., Handyside, A., Hunter, S., and Monk, M. (1987). HPRT-deficient (Lesch-Nyhan) mouse embryos derived from germline colonization by cultured cells. Nature *326*, 292–295.

Incontro, S., Díaz-Alonso, J., Iafrati, J., Vieira, M., Asensio, C.S., Sohal, V.S., Roche, K.W., Bender, K.J., and Nicoll, R.A. (2018). The CaMKII/NMDA receptor complex controls hippocampal synaptic transmission by kinase-dependent and independent mechanisms. Nat. Commun. *9*, 2069.

Ito, I., Hidaka, H., and Sugiyama, H. (1991). Effects of KN-62, a specific inhibitor of calcium/calmodulin-dependent protein kinase II, on long-term potentiation in the rat hippocampus. Neurosci. Lett. *121*, 119–121.

Kopanitsa, M.V., Afinowi, N.O., and Grant, S.G. (2006). Recording long-term potentiation of synaptic transmission by three-dimensional multi-electrode arrays. BMC Neurosci. *7*, 61.

Lavezzari, G., McCallum, J., Lee, R., and Roche, K.W. (2003). Differential binding of the AP-2 adaptor complex and PSD-95 to the C-terminus of the NMDA receptor subunit NR2B regulates surface expression. Neuropharmacology *45*, 729–737.

Leonard, A.S., Bayer, K.U., Merrill, M.A., Lim, I.A., Shea, M.A., Schulman, H., and Hell, J.W. (2002). Regulation of calcium/calmodulin-dependent protein kinase II docking to N-methyl-D-aspartate receptors by calcium/calmodulin and alpha-actinin. J. Biol. Chem. *277*, 48441–48448.

Liu, X.B., Murray, K.D., and Jones, E.G. (2004). Switching of NMDA receptor 2A and 2B subunits at thalamic and cortical synapses during early postnatal development. J. Neurosci. *24*, 8885–8895.

Lowenthal, M.S., Markey, S.P., and Dosemeci, A. (2015). Quantitative mass spectrometry measurements reveal stoichiometry of principal postsynaptic density proteins. J. Proteome Res. *14*, 2528–2538.

Lussier, M.P., Sanz-Clemente, A., and Roche, K.W. (2015). Dynamic regulation of N-methyl-d-aspartate (NMDA) and α-amino-3-hydroxy-5-methyl-4-isoxazolepropionic acid (AMPA) receptors by posttranslational modifications. J. Biol. Chem. *290*, 28596–28603.

Martel, M.A., Ryan, T.J., Bell, K.F., Fowler, J.H., McMahon, A., Al-Mubarak, B., Komiyama, N.H., Horsburgh, K., Kind, P.C., Grant, S.G., et al. (2012). The subtype of GluN2 C-terminal domain determines the response to excitotoxic insults. Neuron *74*, 543–556.

McKay, S., Griffiths, N.H., Butters, P.A., Thubron, E.B., Hardingham, G.E., and Wyllie, D.J. (2012). Direct pharmacological monitoring of the developmental switch in NMDA receptor subunit composition using TCN 213, a GluN2A-selective, glycine-dependent antagonist. Br. J. Pharmacol. *166*, 924–937.

McQueen, J., Ryan, T.J., McKay, S., Marwick, K., Baxter, P., Carpanini, S.M., Wishart, T.M., Gillingwater, T.H., Manson, J.C., Wyllie, D.J.A., et al. (2017). Pro-death NMDA receptor signaling is promoted by the GluN2B C-terminus independently of Dapk1. eLife *6*, e17161.

Mony, L., Zhu, S., Carvalho, S., and Paoletti, P. (2011). Molecular basis of positive allosteric modulation of GluN2B NMDA receptors by polyamines. EMBO J. *30*, 3134–3146.

Nakazawa, T., Komai, S., Tezuka, T., Hisatsune, C., Umemori, H., Semba, K., Mishina, M., Manabe, T., and Yamamoto, T. (2001). Characterization of Fyn-mediated tyrosine phosphorylation sites on GluR epsilon 2 (NR2B) subunit of the N-methyl-D-aspartate receptor. J. Biol. Chem. *276*, 693–699.

Pagano, M.A., Bain, J., Kazimierczuk, Z., Sarno, S., Ruzzene, M., Di Maira, G., Elliott, M., Orzeszko, A., Cozza, G., Meggio, F., and Pinna, L.A. (2008). The selectivity of inhibitors of protein kinase CK2: an update. Biochem. J. *415*, 353–365.

Paoletti, P., Bellone, C., and Zhou, Q. (2013). NMDA receptor subunit diversity: impact on receptor properties, synaptic plasticity and disease. Nat. Rev. Neurosci. *14*, 383–400.

Philpot, B.D., Espinosa, J.S., and Bear, M.F. (2003). Evidence for altered NMDA receptor function as a basis for metaplasticity in visual cortex. J. Neurosci. *23*, 5583–5588.

Prybylowski, K., Chang, K., Sans, N., Kan, L., Vicini, S., and Wenthold, R.J. (2005). The synaptic localization of NR2B-containing NMDA receptors is controlled by interactions with PDZ proteins and AP-2. Neuron *47*, 845–857.

Puddifoot, C., Martel, M.A., Soriano, F.X., Camacho, A., Vidal-Puig, A., Wyllie, D.J., and Hardingham, G.E. (2012). PGC-1α negatively regulates extrasynaptic NMDAR activity and excitotoxicity. J. Neurosci. *32*, 6995–7000.

Qaiser, F., Trembley, J.H., Kren, B.T., Wu, J.J., Naveed, A.K., and Ahmed, K. (2014). Protein kinase CK2 inhibition induces cell death via early impact on mitochondrial function. J. Cell Biochem. *115*, 2103–2115.

Quinlan, E.M., Olstein, D.H., and Bear, M.F. (1999). Bidirectional, experience-dependent regulation of N-methyl-D-aspartate receptor subunit composition in the rat visual cortex during postnatal development. Proc. Natl. Acad. Sci. USA *96*, 12876–12880.

Roche, K.W., Standley, S., McCallum, J., Dune Ly, C., Ehlers, M.D., and Wenthold, R.J. (2001). Molecular determinants of NMDA receptor internalization. Nat. Neurosci. *4*, 794–802.

Rodenas-Ruano, A., Chávez, A.E., Cossio, M.J., Castillo, P.E., and Zukin, R.S. (2012). REST-dependent epigenetic remodeling promotes the developmental switch in synaptic NMDA receptors. Nat. Neurosci. *15*, 1382–1390.

Rutter, A.R., and Stephenson, F.A. (2000). Coexpression of postsynaptic density-95 protein with NMDA receptors results in enhanced receptor expression together with a decreased sensitivity to L-glutamate. J. Neurochem. *75*, 2501–2510.

Ryan, T.J., Emes, R.D., Grant, S.G., and Komiyama, N.H. (2008). Evolution of NMDA receptor cytoplasmic interaction domains: implications for organisation of synaptic signalling complexes. BMC Neurosci. *9*, 6.

Ryan, T.J., Kopanitsa, M.V., Indersmitten, T., Nithianantharajah, J., Afinowi, N.O., Pettit, C., Stanford, L.E., Sprengel, R., Saksida, L.M., Bussey, T.J., et al. (2013). Evolution of GluN2A/B cytoplasmic domains diversified vertebrate synaptic plasticity and behavior. Nat. Neurosci. *16*, 25–32.

Sanz-Clemente, A., Matta, J.A., Isaac, J.T., and Roche, K.W. (2010). Casein kinase 2 regulates the NR2 subunit composition of synaptic NMDA receptors. Neuron *67*, 984–996.

Sanz-Clemente, A., Gray, J.A., Ogilvie, K.A., Nicoll, R.A., and Roche, K.W. (2013). Activated CaMKII couples GluN2B and casein kinase 2 to control synaptic NMDA receptors. Cell Rep. *3*, 607–614.

Sheng, M., Cummings, J., Roldan, L.A., Jan, Y.N., and Jan, L.Y. (1994). Changing subunit composition of heteromeric NMDA receptors during development of rat cortex. Nature *368*, 144–147.

Strack, S., McNeill, R.B., and Colbran, R.J. (2000). Mechanism and regulation of calcium/calmodulin-dependent protein kinase II targeting to the NR2B subunit of the N-methyl-D-aspartate receptor. J. Biol. Chem. *275*, 23798–23806.

Stroebel, D., Carvalho, S., Grand, T., Zhu, S., and Paoletti, P. (2014). Controlling NMDA receptor subunit composition using ectopic retention signals. J. Neurosci. *34*, 16630–16636.

Vieira, M.M., Schmidt, J., Ferreira, J.S., She, K., Oku, S., Mele, M., Santos, A.E., Duarte, C.B., Craig, A.M., and Carvalho, A.L. (2016). Multiple domains in the C-terminus of NMDA receptor GluN2B subunit contribute to neuronal death following in vitro ischemia. Neurobiol. Dis. *89*, 223–234.

Winder, D.G., Martin, K.C., Muzzio, I.A., Rohrer, D., Chruscinski, A., Kobilka, B., and Kandel, E.R. (1999). ERK plays a regulatory role in induction of LTP

by theta frequency stimulation and its modulation by beta-adrenergic receptors. Neuron *24*, 715–726.

Wyllie, D.J., Livesey, M.R., and Hardingham, G.E. (2013). Influence of GluN2 subunit identity on NMDA receptor function. Neuropharmacology *74*, 4–17.

Yanagawa, Y., Kobayashi, T., Ohnishi, M., Kobayashi, T., Tamura, S., Tsuzuki, T., Sanbo, M., Yagi, T., Tashiro, F., and Miyazaki, J. (1999). Enrichment and efficient screening of ES cells containing a targeted mutation: the use of DT-A gene with the polyadenylation signal as a negative selection maker. Transgenic Res. *8*, 215–221.

Zhang, S., Edelmann, L., Liu, J., Crandall, J.E., and Morabito, M.A. (2008). Cdk5 regulates the phosphorylation of tyrosine 1472 NR2B and the surface expression of NMDA receptors. J. Neurosci. *28*, 415–424.

STAR★METHODS

KEY RESOURCES TABLE

REAGENT or RESOURCE	SOURCE	IDENTIFIER
Antibodies		
anti phospho (Ser-1303) GluN2B	Millipore	Cat# 07-398; RRID:AB_310582
anti-phospho (Tyr-1472) GluN2B	Millipore	Cat# AB5403; RRID:AB_177454
anti-phospho (Ser1480) GluN2B	Abcam	Cat# ab73014; RRID:AB_1269572
anti-GluN2B (C terminus)	BD Biosciences	Cat# 610417; RRID:AB_397796
anti-GluN2B (N terminus)	Thermo Fisher Scientific	Cat# 71-8600; RRID:AB_2534001
anti-GluN2A (N terminus)	Thermo Fisher Scientific	Cat# 480031; RRID:AB_2532251
Anti-CamKiiα	Thermo Fisher Scientific	Cat# 13-7300; RRID:AB_2533032
Anti-ERK 1/2	Cell Signaling	Cat# 9102; RRID:AB_330744
Anti-phospho ERK 1/2	Cell Signaling	Cat# 9106; RRID:AB_331768
Anti-Beta actin	Abcam	Cat# ab8227; RRID:AB_2305186
Anti-mouse HRP	Dako	Cat# P0447; RRID:AB_2617137
Anti-rabbit HRP	Cell Signaling	Cat# 7074; RRID:AB_2099233
Normal rabbit IgG	Cell Signaling	Cat# 2729S, RRID:AB_1031062
Chemicals, Peptides, and Recombinant Proteins		
NMDA	Tocris	Cat# 0114/50
Tetrodotoxin citrate (TTX)	Tocris	Cat# 1069/1
(+)-MK801 maleate	Tocris	Cat# 0924/10
(+)-Bicuculline	Tocris	Cat# 0130/50
Picrotoxin	Tocris	Cat# 1128/1G
Spermine	Sigma	Cat# S3256-1G
Ifenprodil hemitartrate	Tocris	Cat# 0545/10
Experimental Models: Organisms/Strains		
Mouse: GluN2A$^{2B(CTR)/2B(CTR)}$	Ryan et al., 2013	N/A
Mouse: GluN2B$^{\Delta CaMKII}$	This paper	N/A
Rat: GluN2A$^{-/-}$	This paper	N/A

CONTACT FOR REAGENT AND RESOURCE SHARING

Further information and requests for resources and reagents should be directed to and will be fulfilled by the Lead Contact, Giles Hardingham (Giles.Hardingham@ed.ac.uk).

EXPERIMENTAL MODEL AND SUBJECT DETAILS

Animals

All animal experiments conformed to national and institutional guidelines including the Animals [Scientific Procedures Act] 1986 (UK), and the Council Directive 2010/63EU of the European Parliament, and had full Home Office ethical approval. All animals were maintained in pathogen-free and light- (12hr light/ 12hr dark) and temperature-controlled conditions. Food and water were available *ad libitum.* Animals were group-housed in conventional cages and were provided with environmental enrichment. Animals had not been subject to previous procedures. P14 and P28 homozygous and wild-type mice of both sexes were used and were generated as a result of heterozygous intercrosses to generate littermate controls. GluN2A$^{2B(CTR)/2B(CTR)}$ and GluN2B$^{\Delta CaMKII}$ mice were maintained on a C57BL/6J background. GluN2A$^{-/-}$ rats were maintained on a Long Evans Hooded background, and E20.5 rats of all genotypes were generated as a result of heterozygous intercrosses.

Primary cultures

Cortical mouse and rat neurons were cultured as described (Puddifoot et al., 2012) at a density of 9-13 × 10^4 neurons per cm^2 from E17.5 mice or E20.5 rats (mixed sexes) with Neurobasal growth medium supplemented with B27 (Invitrogen, Paisley, UK). The

cultures were maintained at 37°C with 5% CO2. Experiments were performed at DIV 8-18 as indicated. By DIV 8 the neurons are capable of supporting synchronous synaptic activity, but NMDA receptors are primarily GluN2B-containing, whereas at DIV 18 there is a substantial GluN2A component (Baxter et al., 2015; McKay et al., 2012).

Generation of the GluN2B$^{\Delta CaMKII}$ and mouse

GluN2A$^{2B(CTR)/2B(CTR)}$ neurons (Ryan et al., 2013) were produced from GluN2A$^{2B(CTR)/2B(CTR)}$ embryos resulting from GluN2A$^{WT/2B(CTR)}$ x GluN2A$^{WT/2B(CTR)}$ mating, which also yielded GluN2A$^{WT/WT}$ littermate controls. Specific details regarding the generation of GluN2A$^{2B(CTR)}$ line can be found elsewhere (Ryan et al., 2013). The GluN2A^{WT} allele was genotyped using primers (5′-CTTCTTTTCTTCAATGTGCACTCC −3′) and (5′-CTACATCATCAGAAGCCCACC −3′) while the GluN2A$^{2B(CTR)}$ allele was genotyped using primers (5′-GGGGAAGTTACATGGTGGATTG-3′) and (5′-GGGATGATCAGTGCTTGCTTC-3′).

Generation of the GluN2B$^{\Delta CaMKII}$ mouse

An artificial polylinker containing Xho1 and HindIII restriction sites was inserted into the NotI and SalI sites of pSP72 (Promega), resulting in pSP72-L. A Diphtheria Toxin A (DT-A) fragment driven by an MC1 promoter with a mouse SV40 polyadenylation signal was digested with HindIII and XhoI, and inserted into the HindIII and SalI sites of pSP72-L, creating pSP72-L-DT-ApA (Yanagawa et al., 1999). A previously generated GluN2B targeting vector was acquired and co-opted for construction of the GluN2B-CaMKII vector (Delint-Ramirez et al., 2010). The GluN2B-CaMKII targeting vector included a neomycin phosphotransferase gene (Neo) driven by a compound phosphoglycerate kinase (PGK) and EM7 promoter for kanamycin resistance in bacteria, and G418 resistance in murine cells, respectively. Substitutions were introduced into the GluN2B 3′ terminal exon by mutagenic PCR using primers, resulting in the GluN2B-CaMKII vector. The GluN2B-CaMKII vector was then inserted into the NotI and XhoI sites of pSP72-L-DT-ApA, generating the final GluN2B S1303D targeting vector. The targeting vector was linearized with NotI and electroporated into E14Tg2A (129/OlaHsd) mouse embryonic stem (ES) cells (Hooper et al., 1987). ES cells colony selection was carried out using G418. Genomic DNA was extracted from individually picked ES cell colonies and used for screening for targeting vector integration by long-range PCR using primers P1 (ACGAGATCAGCAGCCTCTGTTCCAC) and P2 (GAGGCGGGGCGACCAGGAAGGC). P1, hybridizes to the Neo cassette, P2 hybridizes to a region of genomic DNA that is 3′ of the targeting vector 3′ homology arm. The GluN2B C-terminal exon was cloned from targeted ES cell lines and sequenced to confirm the presence of the mutated sites (data not shown). ES cell clones were injected into C57BL/6J mouse blastocysts, which were then implanted into a pseudopregnant mouse. Chimeras were identified from the offspring based on coat color. Male chimeras were crossed onto C57BL/6J females in order to obtain an F1 generation. Genomic DNA was extracted from F1 progeny and used for confirmation of germline transmission by PCR using primers P1 and P2, and primers P3 (TCAGTGCTTGCTTCACGGCAGC) and P4 (CTCCTCTCCAGCCTCCCACACT). P3 hybridizes to the GluN2B C-terminal domain exon, and P4 to the NR2B 3′ UTR. F1 heterozygotes were crossed onto transgenic mice expressing Cre recombinase under a CMV promoter to excise the Neo selection cassette. Neo removal in the F2 generation was assessed by PCR using primers P3 and P4, which gives a 567 bp product for wild-type mice and a 628 bp product for mutant alleles without the Neo cassette, and P4 and P5 (TGGAAGGATTGGAGCTACGGG), which gives a ~700 bp product only if the Neo cassette is present. Mice were genotyped using primers P3 and P4. The line was further backcrossed onto C57BL/6J for 6 generations prior to the commencement of experiments. Animals were treated in accordance with UK Animal Scientific Procedures Act (1986). All the experiments were performed using wild-type and homozygous littermates.

Generation of the GluN2A$^{-/-}$ rat

Single cell Long Evans Hooded rat embryos underwent pronuclear microinjection of mRNA encoding the enzyme Cas9 and small guide RNAs (sgRNA) binding to 5′ and 3′ of exon 8 of Grin2A, before being implanted into pseudopregnant mothers. The resulting live births were screened by PCR for genomic deletions due to repair by non-homologous end joining of double stranded breaks targeted to either side of exon 8. A 1065bp deletion spanning exon 8 (which encodes key pore forming domains of GluN2A) was identified, and confirmed by sequencing (data not shown). Genotyping was performed using primer pairs P5 (AGGGAAGAAGGGAA CAGGAG) with P6 (TCTCTGGGATTCAGTGCAGA) and P7 (AAGGCAGAGAGAGAGACAAAG) with P8 (ATGGCAGTTCCCAGTA GCAT). P5 and P7 bind to 5′ of the deletion, P6 binds to 3′ of the deletion, and P8 binds within the deletion. The sgRNA design and generation of the founder animals were performed by Horizon Discovery Group plc (St Loius, MO, USA). Animals were treated in accordance with UK Animal Scientific Procedures Act (1986). All the experiments were performed using wild-type, heterozygous, and homozygous littermates matched animals.

METHOD DETAILS

Cell culture electrophysiological recording and analysis

Whole cell patch clamp recordings were performed as described (Edman et al., 2012; Hardingham et al., 2007). Briefly, coverslips containing cortical neurons were transferred to a recording chamber perfused (at a flow rate of 3-5 ml/min) with an external recording solution composed of (in mM): 150 NaCl, 2.8 KCl, 10 HEPES, 2 $CaCl_2$, 10 glucose and 0.1 glycine, pH 7.3 (320-330 mOsm). Tetrodoxin (300 nM) was included to block action-potential driven excitatory events. Patch-pipettes were made from thick-walled borosilicate glass (Harvard Apparatus, Kent, UK) and filled with a K-gluconate-based internal solution containing (in mM): potassium gluconate 141, NaCl 2.5, HEPES 10, EGTA 11; pH 7.3 with KOH). Electrode tips were fire-polished for a final resistance ranging between 3-5 MΩ. All NMDA currents were evoked by 150 μM NMDA and 100 μM glycine, bath applied using a perfusion system. Currents were recorded at room temperature (21 ± 2°C) using an axopatch 200B amplifier (Molecular Devices, Union City, CA).

Neurons were voltage-clamped at –60 mV and recordings were rejected if the holding current was greater than –100 pA or if the series resistance drifted by more than 20% of its initial value (< 20 MΩ). Whole-cell currents were analyzed using WinEDR v3.2 software (John Dempster, University of Strathclyde, UK).

To measure extrasynaptic NMDAR currents, synaptically-located NMDARs were blocked by the use-dependent antagonist MK-801, in an activity-dependent manner as previously described (Martel et al., 2012). First, whole-cell NMDAR currents were recorded in voltage-clamp (150 μM NMDA, in Mg^{2+}-free and TTX containing recording solution). Neurons were then switched to current-clamp mode in normal Mg^{2+}-containing, TTX-free medium in the presence of MK-801 (10 μM) and bicuculline (50 μM). After 5 minutes of neuronal firing, the neurons were switched back to voltage clamp and the whole-cell NMDA currents were re-assessed as above; the slow off-rate of MK-801 ensures synaptic NMDARs remained blocked and only extrasynaptic NMDAR currents are recorded.

To determine the ifenprodil-sensitivity of neurons, whole cell NMDA currents were recorded followed by the inclusion of 3 μM ifenprodil in the recording solution for a blocking period of 90 s. The whole cell NMDA current was the re-assessed, with 3 μM ifenprodil included, and the % block calculated.

For determining spermine potentiation, neurons were initially voltage-clamped at −60mV using the internal solution and ACSF described above. The neurons were then voltage clamped at −30 mV and switched to a recording solution composed of (in mM): 70 NaCl, 60 choline chloride, 2.8 KCl, 20 HEPES, 10 glucose, 0.1 glycine, pH 6.5. 100 μM pentetic acid was also included otherwise rapid desensitization of the spermine potentiation occurred. NMDA currents were evoked (as described above) then potentiated by 100 μM and 200 μM spermine consecutively. 200 μM spermine was originally included as an internal control: both 100 μM and 200 μM spermine are on the linear part of the dose-response and an approximate 2-fold increase in potentiation was expected jumping from 100 μM to 200 μM spermine.

Slice electrophysiological recordings and analysis

For extracellular and EPSC recordings illustrated in Figures 2D–2I, S2B, and S2C, mice were deeply anaesthetised with halothane and then sacrificed by cervical dislocation using techniques approved by UCLA Institutional Animal Care and Use Committee. Following the dissection of the hippocampi from the rest of the brain, 400 μm thick hippocampal slices were cut using a manual tissue chopper and then maintained at 30°C in an interface-slice type recording chamber perfused with oxygenated (95% O_2/5% CO_2) artificial cerebrospinal fluid (ACSF) containing 124 mM NaCl, 4.4 mM KCl, 25 mM $NaHCO_3$, 1 mM NaH_2PO_4, 2 mM $CaCl_2$, 1.2 mM $MgSO_4$, and 10 mM glucose. Whole-cell voltage-clamp techniques were used to record AMPA receptor- and NMDA receptor-mediated excitatory postsynaptic currents (EPSCs). In these experiments, slices were bathed in a modified ACSF containing 4.0 mM $CaCl_2$, 4.0 mM $MgSO_4$, 2.4 mM KCl, and 100 μM picrotoxin. The CA3 region was removed to prevent bursting in the absence of inhibition and slices were maintained at 30°C in a submerged slice recording chamber. Recording electrodes (3–6 MOhms) were filled with a solution containing 102 mM cesium gluconate, 17.5 mM CsCl, 10 mM TEA-Cl, 5 mM QX314, 4.0 mM Mg-ATP, 0.3 mM Tris-GTP, and 20 mM HEPES (pH = 7.2). EPSCs evoked by Schaffer collateral fiber stimulation at 0.2 Hz were recorded at membrane potentials of −80 mV or +40 mV, and the AMPA receptor- and NMDA receptor-mediated components of the synaptic currents were estimated by measuring EPSC amplitude 5 and 50 ms after EPSC onset, respectively. In these experiments, the intensity of presynaptic fiber stimulation was adjusted to elicit EPSCs with peak amplitudes of approximately 200 pA at −80 mV. Spontaneous miniature EPSCs were recorded at −80 mV in the presence of 1.0 μM TTX. A combination of template and threshold (6 pA) based event detection routines in pClamp 10 (Molecular Devices) was used for mEPSC analysis. Extracellular recordings (Figures S2B and S2C) were done under interface conditions and began after allowing the slices to recover for at least 1 hour. In the experiments illustrated in Figures 2J, 2K, and S2A, fEPSPs were recorded by using microelectrode arrays (MEA60, Multi Channel Systems, Reutlingen, FRG). Input/output relationships and theta-burst stimulation-induced LTP were examined using hippocampal slice preparations and experimental conditions described in detail previously (Kopanitsa et al., 2006). Theta-burst stimulation consisted of 10 bursts of 100 Hz stimulation (4 pulses) delivered with an inter-burst interval of 200 ms. The magnitude of LTP present 60–65 minutes post theta-burst stimulation was used for statistical comparisons.

PSD fractionation, co-immunoprecipitation and western blotting

To isolate PSD-enriched proteins (containing synaptic NMDARs) and we used an approach previously described for studying synaptic and extrasynaptic NMDAR populations (Martel et al., 2012). Briefly, brains from P14 and P28 mice were dissected, their cortices removed and immediately placed in ice-cold homogenization buffer (10 mM Sucrose, 10 mM HEPES, supplemented with Protease and Phosphatase Inhibitor Cocktail tablets (Roche, Burgess Hill, UK), pH 7.4). Tissue samples were homogenized with a teflon/glass homogenizer then centrifuged (1,000 g, 10 min, 4°C). The supernatant was collected and centrifuged at 12,000 g (20 min, 4°C), after which the pellet was resuspended twice in 4mM HEPES (containing 1mM EDTA, pH 7.4) by repeating the last centrifugation step. To obtain the non-PSD enriched fraction, pellets were then resuspended in 20 mM HEPES, 100 mM NaCl and 0.5% Triton X-100 (pH 7.2). Samples were incubated 15 min at 4°C while rotating gently, followed by centrifugation (12,000 g, 20 min, 4°C). The supernatant was collected (Non-PSD enriched fraction) and the pellet solubilized (in 20 mM HEPES, 0.15 mM NaCl, 1% Triton X-100, 1% sodium deoxycholate (DOC), 1% SDS, 1 mM DTT, pH 7.5) for a further 1h at 4°C with gentle agitation. Finally, the samples were centrifuged at 10,000 g for 15 min (4°C) and the supernatants were collected as PSD-enriched fractions. Fractions were stored at −20°C until western blotting, and used less than two weeks after preparation.

For co-immunoprecipitation, P28 cortices were homogenized in IP buffer (50 mM Tris HCl pH 9, 50 mM NaF, 20 μM $ZnCl_2$, 1 mM Na_3VO_4, 1:100 protease inhibitor cocktail set III (Merck Chemicals, Nottingham, UK), 0.5 mg/ml PMSF, 1% DOC) and centrifuged for 40 min at 25000 g. The protein concentration in the supernatant was measured (by BCA assay) and adjusted to 3.5 mg/ml using IP buffer. Antigen binding was carried-out by adding of 2 μg of antibody (anti-GluN2B (N terminus,), or control IgG) to 1.75 mg of protein extract. The antibody-antigen mixture was incubated overnight with rotation at 4°C. 50 μl Dynabeads® were used per immunoprecipitation reaction incubation, and were aliquoted to each antigen-antibody mixture. Beads were incubated with the antibody for 20 min at room temperature on a rotator. The Dynabead-antibody-antigen complex was then washed four times in IP buffer, before being eluted by suspending the beads in 40 μL of 1.5x 40 μL of 1.5x LDS sample buffer (NuPage, Life Technologies) and boiling for 10 min. The supernatant was then stored at −80°C until SDS-PAGE and western blotting was carried out.

For western blotting, a procedure similar to that previously described was employed (Baxter et al., 2011). In order to minimize the chance of post-translational modifications during the harvesting process, neurons were lysed immediately after stimulation in 1.5x LDS sample buffer (NuPage, Life Technologies) and boiled at 100°C for 10 min. Approximately 10 μg of protein was loaded onto a precast gradient gel (4%–12%) and subjected to electrophoresis. Briefly, western blotting onto a PVDF membrane was then performed using the Xcell Surelock system (Invitrogen) according to the manufacturer's instructions. Following the protein transfer, the PVDF membranes were blocked for 1 hour at room temperature with 5% (w/v) non-fat dried milk in TBS with 0.1% Tween 20. The membranes were incubated at 4°C overnight with the primary antibodies diluted in blocking solution: anti phospho-(Ser-1303) GluN2B (1: 2000, Millipore), anti-phospho (Tyr-1472) GluN2B (1:2000, Millipore), anti-phospho (Ser1480) GluN2B (1:2000, Abcam), anti-GluN2B (C terminus, 1:4000, BD Transduction Laboratories), anti-GluN2B (N terminus 1:2000, Thermo Fisher Scientific), anti-GluN2A (N terminus, 1:1000, Thermo Fisher Scientific), anti-CamKiiα (1:8000, Thermo Fisher Scientific), anti-ERK1/2 (1:2000, Cell Signaling), anti-phospho ERK1/2 (1:2000, Cell Signaling) and anti-beta actin (1:200000, Abcam). For visualization of western blots, HRP-based secondary antibodies were used followed by chemiluminescent detection on Kodak X-Omat film. Western blots were digitally scanned and densitometric analysis was performed using ImageJ. All analysis involved normalizing to either GluN2B (for phospho-GluN2B antibodies) or beta actin expression as a loading control.

Transfection of cortical neurons

Neurons were transfected at DIV 7 using Lipofectamine 2000 (Invitrogen) according to the manufacturer's suggested protocol as previously described (Bell et al., 2015). β-globin or pCis-GluN2A (Rutter and Stephenson, 2000) were co-transfected with enhanced green fluorescent protein (eGFP), to identify transfected cells, in a ratio of 2:1. Transfection efficiency was approximately 5% with > 99% of eGFP-expressing cells being identified as positive for the neuronal nuclear antigen (NeuN), while < 1% were positive for glial fibrillary acidic protein (GFAP) (Hasel et al., 2017). Electrophysiological recordings were made from transfected neurons 72-96 h post transfection.

QUANTIFICATION AND STATISTICAL ANALYSIS

Statistical analysis, equipment and settings

Statistical testing involved a 2-tailed paired Student's t test., or a one- or two-way ANOVA followed by Sidak's post hoc test. Cell death analyses for both *in vitro* and *in vivo* experiments were performed blind to the genotype/experimental condition. For western blots, we used chemiluminescent detection on Kodak X-Omat film, and linear adjustment of brightness/contrast applied (Photoshop) equally across the image, maintaining some background intensity. In some cases, lanes from non-adjacent lanes are spliced together, but the lanes are always from the same blot, processed in the same way, and the splicing point is clearly marked. For the analysis of electrophysiological data in MEA-based recordings (Figures 2J, 2K, and S2A), because several slices were routinely recorded from every mouse, the values of the area under the I/O relationship ($AUC_{I/O}$), values of peak fEPSP amplitudes evoked by maximum stimulus strength and LTP in WT and mutant mice were compared by the two-way nested ANOVA with genotype (group) and mice (sub-group) as fixed and random factors, correspondingly, with the Satterthwaite's correction applied to calculate effective degrees of freedom (STATISTICA v. 10, StatSoft, Inc., Tulsa, OK, USA). Statistical effects were considered significant if $p < 0.05$. Statistical details of each experiment can be found within figure legends.

Cell Reports, Volume *25*

Supplemental Information

The Developmental Shift of NMDA Receptor Composition Proceeds Independently of GluN2 Subunit-Specific GluN2 C-Terminal Sequences

Sean McKay, Tomás J. Ryan, Jamie McQueen, Tim Indersmitten, Katie F.M. Marwick, Philip Hasel, Maksym V. Kopanitsa, Paul S. Baxter, Marc-André Martel, Peter C. Kind, David J.A. Wyllie, Thomas J. O'Dell, Seth G.N. Grant, Giles E. Hardingham, and Noboru H. Komiyama

Supplemental Figures

Fig. S1

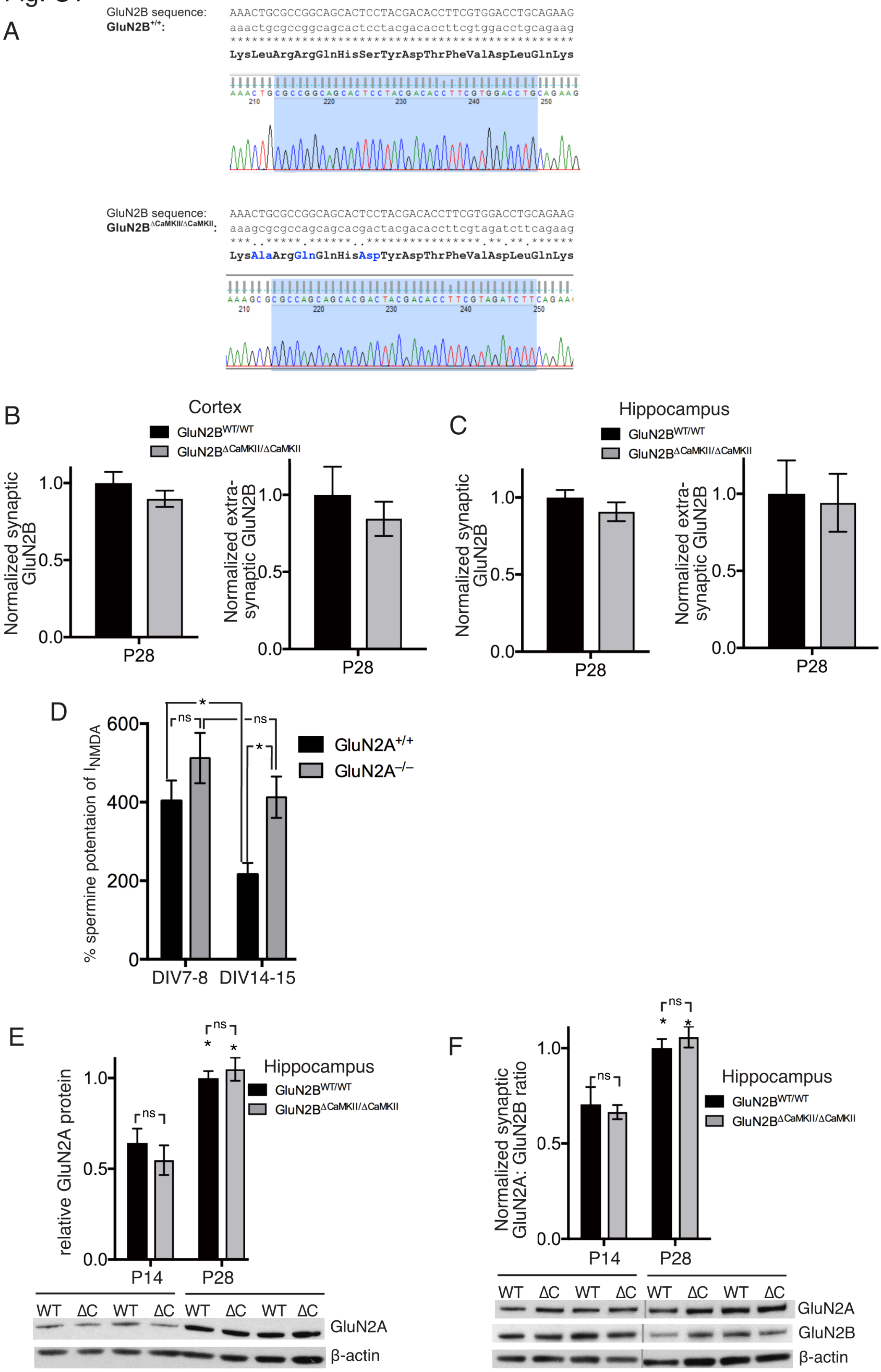

Fig. S1, relating to Figure 1. A) Sequencing data showing the mutations knocked into the *Grin2b* gene in the GluN2B$^{\Delta CaMKII/\Delta CaMKII}$ mouse line. **B)** Synaptic (left) and extrasynaptic (right) GluN2B protein expression was measured in P28 neocortices of GluN2B$^{\Delta CaMKII/\Delta CaMKII}$ (n=8) and GluN2B$^{+/+}$ mice (n=8). **C)** Synaptic (left) and extrasynaptic (right) GluN2B expression was measured in P28

hippocampi of GluN2B$^{\Delta CaMKII/\Delta CaMKII}$ (n=8) and GluN2B$^{+/+}$ mice (n=8). **D)** Developmental shift in spermine potentiation of NMDAR currents requires GluN2A. Percentage potentiation of NMDAR currents by spermine (200 µM) was measured at the indicated stages for GluN2A$^{+/+}$ and GluN2A$^{-/-}$ rat neurons. *P<0.05 compared to DIV7-8 of the same genotype, 2-way ANOVA plus Sidak's post-hoc test (n=12 of all ages/genotypes). **E)** Whole neocortical extracts from P14 and P28 mice of the indicated genotype were analysed for GluN2A expression, normalized to ß-actin. *P<0.05 compared to P14 of the same genotype, 2-way ANOVA plus Sidak's post-hoc test (n=8). Lower panel shows example blot. **F)** Post-synaptic density (PSD) extracts from P14 and P28 mice of the indicated genotype were analysed for GluN2A and GluN2B expression, normalized to ß-actin, and the ratio calculated. *P<0.05 compared to P14 of the same genotype, 2-way ANOVA plus Sidak's post-hoc test (n=8). Lower panel shows example blot.

Fig. S2

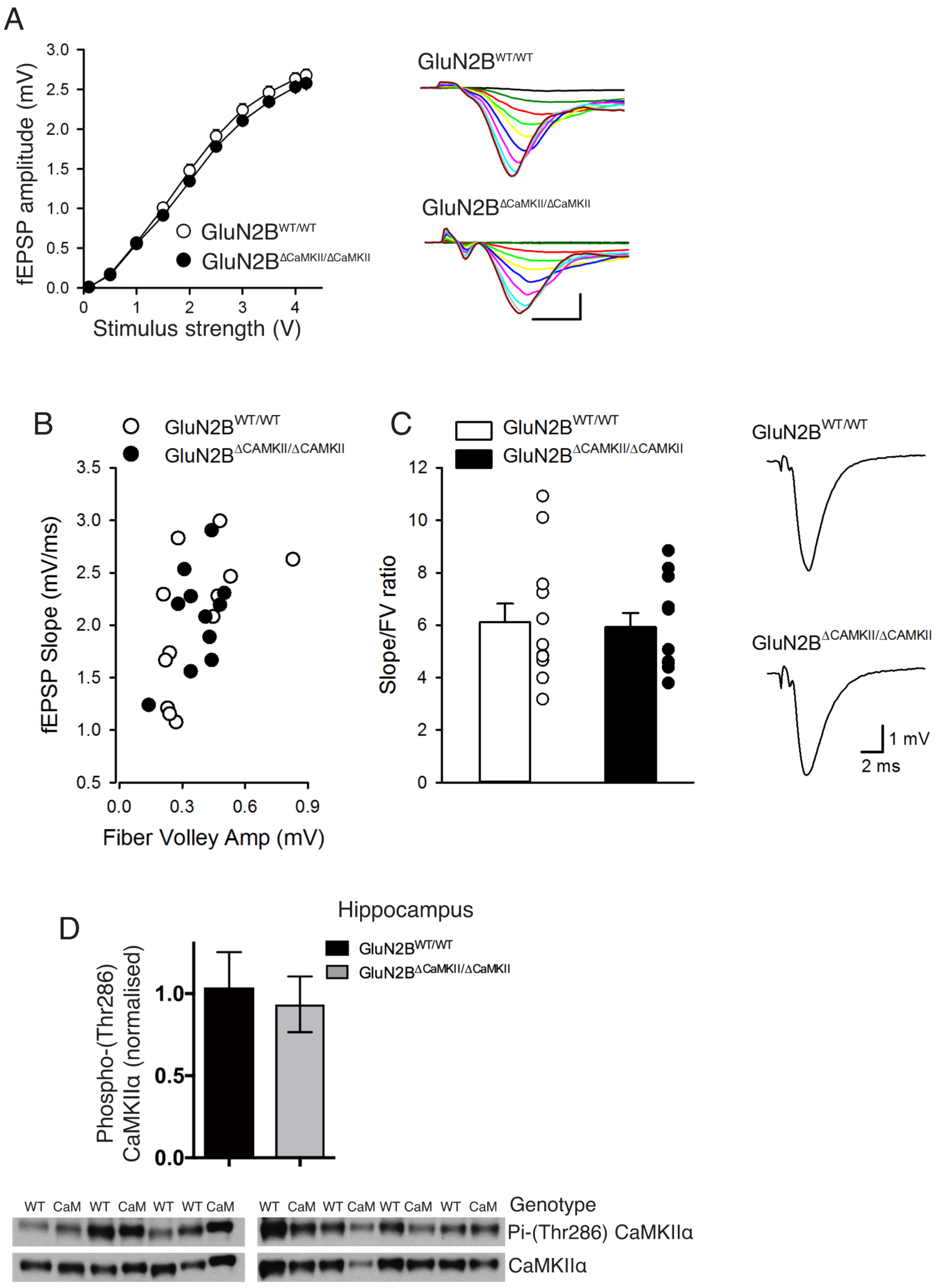

Fig. S2, relating to Figure 2. A) Input-output relationships illustrate averaged fEPSP amplitudes in slices from GluN2B$^{\Delta CaMKII/\Delta CaMKII}$ (35 slices from n=11 mice) and GluN2B$^{WT/WT}$ mice (29 slices from n=9 mice) in response to stimulation of Schaffer collaterals by biphasic voltage pulses of 0.1 – 4.2 V. Right panel shows representative families of fEPSP traces. Scale bar: 1 mV/2 ms. **B-C)** A comparison of fEPSP slopes and presynaptic fiber volley amplitudes for 12 slices from 5 GluN2B$^{WT/WT}$ mice and 11 slices from 6 GluN2B GluN2B$^{\Delta CaMKII/\Delta CaMKII}$ mice. **D)** Phospho-T286 CaMKIIα levels in PSD fractions of P28 GluN2B$^{\Delta CaMKII/\Delta CaMKII}$ vs GluN2B$^{WT/WT}$ hippocampi were analysed by western blot, normalising to total CaMKIIα.

Fig. S3

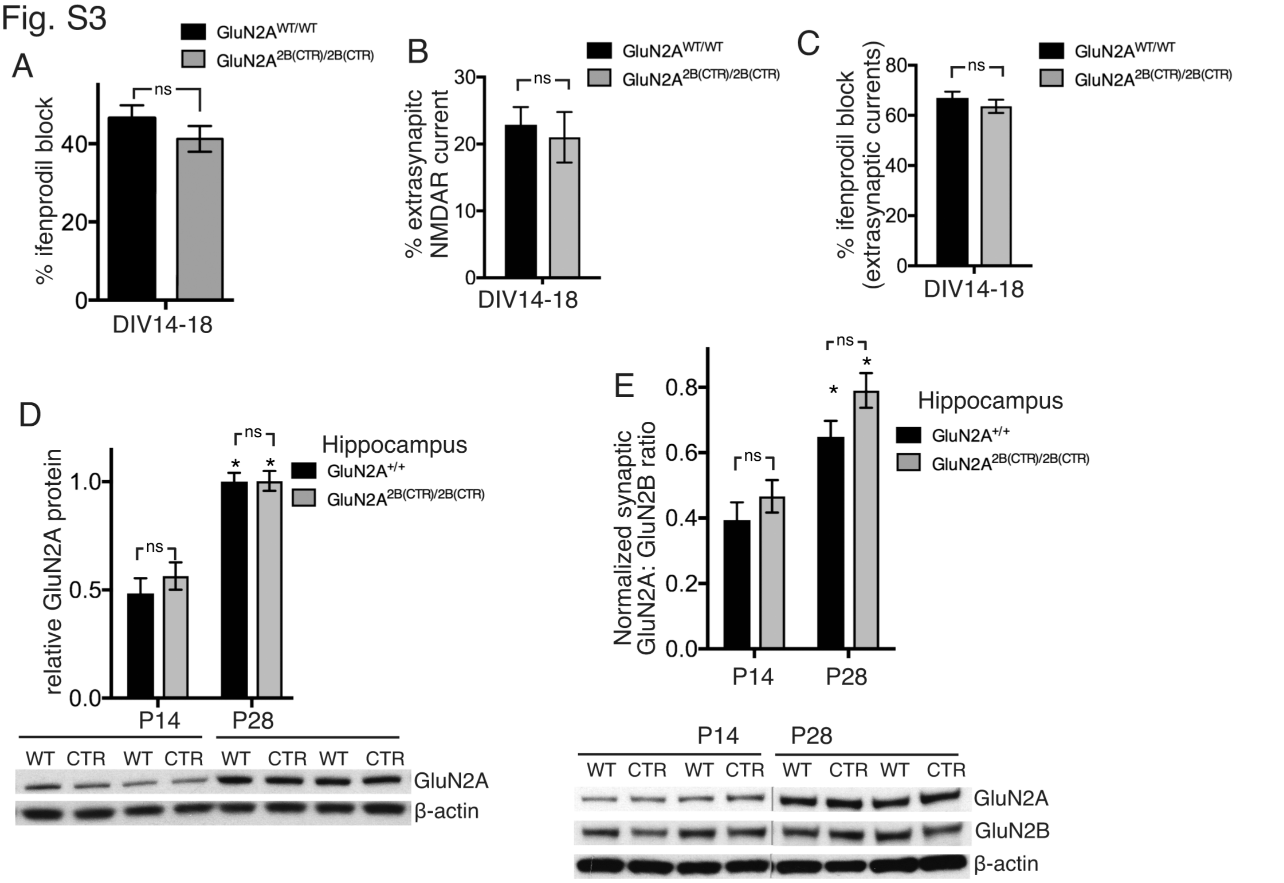

Fig. S3, relating to Figure 3. A) Ifenprodil sensitivity of NMDAR currents was measured at DIV14-18 in neurons of the indicated genotypes. GluN2A$^{WT/WT}$: n=24 cells; GluN2A$^{2B(CTR)/2B(CTR)}$: n=30 cells. **B,C)** Extrasynaptic NMDAR currents were calculated and expressed as a percentage of whole-cell currents (B) and their sensitivity to ifenprodil (3 μM) was measured in DIV14-18 neurons, an age where significant GluN2A is expressed. N=12 of both genotypes. **D)** Whole hippocampal extracts from P14 and P28 mice of the indicated genotype were analysed for GluN2A expression, normalized to ß-actin. *P<0.05 compared to P14 of the same genotype, 2-way ANOVA plus Sidak's post-hoc test (n=8). **E)** Post-synaptic density (PSD) extracts from P14 and P28 hippocampi of the indicated genotype were analysed for GluN2A and GluN2B expression, normalized to ß-actin, and the ratio calculated. *P<0.05 compared to P14 of the same genotype, 2-way ANOVA plus Sidak's post-hoc test (n=8).
